# An Inorganic Chemistry Laboratory Technique Course using Scaffolded, Inquiry-Based Laboratories and Project-Based Learning

**DOI:** 10.1021/acs.jchemed.3c00547

**Published:** 2023-08-15

**Authors:** Chun Chu, Jessica L. Dewey, Weiwei Zheng

**Affiliations:** † Department of Chemistry, Syracuse University, Syracuse, New York 13244, United States; ‡ Duke Learning Innovation, Duke University, Durham, North Carolina 27708, United States

**Keywords:** Upper-Division Undergraduate, Scaffolded Lab Experiments, Project-Based Learning, Inorganic Chemistry, Problem-Solving Skills

## Abstract

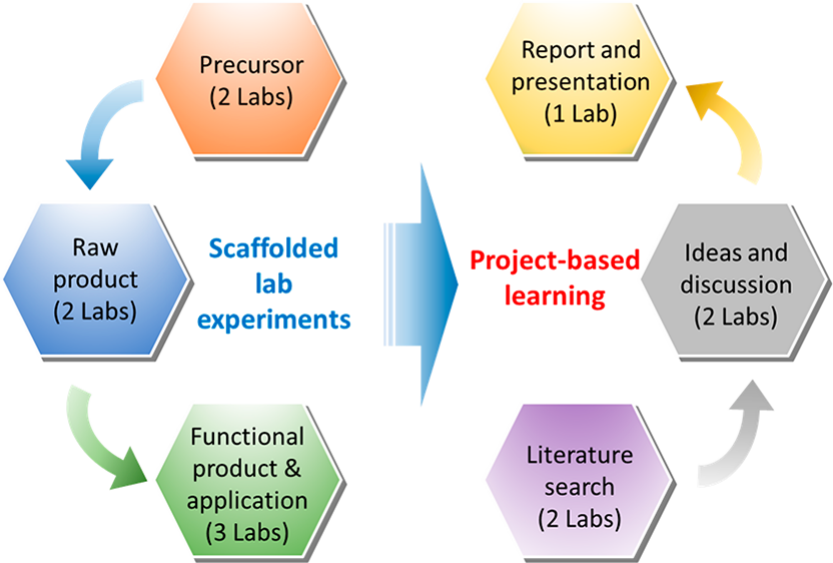

To enhance students’ learning and help them understand the whole picture of the field of inorganic chemistry, an inorganic laboratory technique course was designed that uses scaffolded, inquiry-based lab experiments and project-based learning. The scaffolded, inquiry-based laboratories taught in the first 8 weeks of the course helped students better understand the aim of each lab and how to apply each lab technique to a bigger research project. The laboratory experiments also included opportunities for cooperative and collaborative learning through student group work and feedback. To further develop students’ independent research skills, we implemented project-based learning in the second part of the course (last 4 weeks), in which students develop a research proposal based on independent literature research and the laboratory techniques they learned from the course. Pilot data suggest that the course helped improve students’ interest in inorganic chemistry, science self-efficacy, and science identity. Additionally, students reported that both the scaffolded, inquiry-based laboratories and the project-based learning module enhanced their problem-solving and critical thinking skills.

## Introduction

As an upper-level course, the inorganic chemistry laboratory provides a great opportunity for students to learn and practice the skills and knowledge of chemistry by *performing* inorganic chemistry work.^1^ However, inorganic chemistry, unlike some other areas of chemistry (e.g., general and organic chemistry), generally contains a wide breadth of topics and methodologies which require significant effort for students to understand, especially if they are not clearly connected.^1,2^ Additionally, while there have been recent calls and renewed interest in incorporating inquiry-based learning into chemistry lab courses given the potential benefits of increased science literacy, research skills, and sense of scientific ability,^3−7^ results from a recent national survey indicate that the majority of inorganic chemistry laboratory experiments involve minimal opportunities for inquiry.^1^ Inorganic chemistry courses that cover a broad range of topics across individual laboratories that do not relate to, or build upon, each other and are often taught without opportunities for inquiry may therefore fail to pique the interest of undergraduate students and make it difficult for students to understand and apply their knowledge of chemistry.^8,9^

The level of inquiry in a lab course can be characterized by determining how much guidance is provided to students throughout an experiment, and which aspects of the experiment are given to students.^10^ More specifically, the characteristics of a lab experiment include the problem/question, theory/background, procedures/design, results analysis, results communication, and conclusions.^10^ As students are given more opportunities for independence and are provided with fewer answers (e.g., not given the conclusions of an experiment or told how to communicate the results), the level of inquiry increases, making the experience more similar to the authentic scientific research process.^11,12^ The use of inquiry-based laboratories (laboratories that ask students to use methods and practices that would be used by professional scientists when constructing new knowledge) has been shown to improve students’ interest in and understanding of science.^11,13−15^ Additionally, inquiry-based lab experiments can demonstrate the real conditions and imperfection of chemical reactions, which provide students with an opportunity to develop their critical thinking and problem-solving skills.^14,15^ Furthermore, in place of the many disconnected, cookbook-style experiments taught in traditional lab courses, the incorporation of scaffolding, either through an overarching theme or by purposefully designing laboratories to build on each other, may also support students’ engagement and perceived success in a course.^1,16^

One additional approach that has been used to improve student learning and engagement in chemistry lab courses is project-based learning (PBL).^17−19^ PBL organizes learning around a realistic project driven by students’ interests.^19^ Students engage in an extended inquiry process structured around a complex, authentic question, and both learn and apply content and skills simultaneously.^18,20^ The PBL approach is flexible in that it can be adapted to various timeframes during a semester and can be catered to the specific contexts in which students are learning. Additionally, the opportunity for students to design a project based on their own interests supports students’ need for autonomy and can ultimately help improve their intrinsic motivation when it comes to inorganic chemistry.^21,22^

The inorganic chemistry laboratory technique course described here uses both scaffolded, inquiry-based laboratories and project-based learning. The inquiry-based laboratories designed for this course can be characterized as “guided inquiry”.^10^ Students are provided with the problem/question, theory/background, and procedures of each lab; however, each lab has an unknown or broad range of results and the students must interpret and analyze the data, develop their own conclusions, and communicate their findings.^10^ These laboratories are also scaffolded, meaning they relate to, and build upon, each other under the overarching theme of inorganic materials. Project-based learning was then used to provide students with the opportunity to apply what they had learned from the inquiry-based laboratories and develop their skills in identifying research questions, finding related theory/background information, and designing relevant experiments. In the present work, the PBL approach is used to focus on the structural, optical, and electronic properties, as well as the applications of inorganic materials. Ultimately the choice to use both scaffolded, inquiry-based laboratories and project-based learning ensured that students had a chance to learn and practice all of the skills that are fundamental for inorganic chemistry.^1^

## Course Overview

For many years, Syracuse University did not have a standalone inorganic chemistry laboratory course. Instead, upper-level students needing or wanting to take an inorganic chemistry lab were grouped into the honors general chemistry lab II course with freshmen. However, this course mainly covered biochemistry experiments and did not include many inorganic chemistry experiments. To expand the inorganic chemistry curricula and provide undergraduate students with hands-on experience of the synthesis and characterization of a variety of inorganic compounds and nanocrystals, we designed a new Inorganic Chemistry Laboratory Technique course in Spring 2020 that incorporated scaffolded, inquiry-based experiments along with project-based learning. The scaffolded, inquiry-based laboratories are taught over the first 8 weeks of the course and are designed specifically so that later laboratories are based on, and build from, the earlier laboratories. For example, we start by making molecular precursors at the beginning of the semester; then we use the precursors to synthesize inorganic nanoparticles. It should be noted that a broad range of sizes and compositions (in the case of core/shell) of the inorganic nanoparticles could be obtained from the experiments, potentially leading to the largely unknown optical properties of the as-synthesized products due to the unique size-, composition-, and surface defect-dependent optical properties of materials in nanoscale.^23,24^ We also designed three laboratories focusing on the study of the stability, optical, and structural properties of the molecular precursors and nanoparticles using three basic characterization methods, thermogravimetry (TGA), optical spectroscopies (UV–vis absorption and emission), and X-ray diffraction (XRD), that are routinely used in chemistry research laboratories. We used this approach to help students better understand the aim of each lab, how to apply these aims to a bigger research project, how to interpret data, and how to explain results based on previous laboratories/observations. The nature of the inquiry-based experiments is similar to projects developed in the instructor’s research group in the past few years through the Research Experiences for Undergraduates (REU) program. In the experiments, we chose to synthesize and characterize nanoparticles, a new type of inorganic material, to show students the state-of-the-art and dynamic nature of research in chemistry.

To further build on and develop the students’ research skills, in the second part of the lab course, we developed a four week project-based learning module in which students develop a research proposal based on the laboratory techniques they learned from the course and their own independent literature research. Students choose their own topic and research question, propose a research design for how they might address their question, and outline the expected results from their proposed design. Students both write a project summary and make an oral presentation of their project to their classmates at the end of the semester (see an example student presentation in Supporting Information). This module gives them the authentic experience of proposing and designing an experiment to answer a research question, which can help increase their interest and motivation in the content and also helps develop professional scientific communication skills.

## Description of Student Population

The students enrolling in this course are typically chemistry majors in their junior or senior year. They generally completed the inorganic chemistry lecture course. However, the lecture class is not a prerequisite or corequisite for this laboratory class, and the material in the laboratory class is independent of the lectures. Over the four offerings of this course, the number of students taking the class has ranged from 7 to 13. The registered students are distributed between two sections that meet on different days of the week. The lab space is equipped with six standard fume hoods. Thus, a maximum of six students for each section may be ideally accommodated by the course organization described here.

## Course Outline and Description

Compared to traditional chemistry lab courses, this new course contains a unique combination of scaffolded, inquiry-based experiments taught in Part 1 of the course (8 weeks) and a PBL module in Part 2 of the course (4 weeks). Each lab lasts 3 h, and the general format of each lab includes an instructor’s lecture at the beginning of each lab that presents the concepts of the lab experiment as well as a specific lecture related to research skills (∼45 min), followed by the set up and completion of the lab experiment. For laboratories 2 and 6, which involved significant instrument acquisition time (≥30 min per measurement), the TA and students first set up their experiment and then move to the lecture and discussion to minimize the waiting time during the lab courses. Table 1 presents a timeline of all of the experiments students work on during the semester.

**Table 1:** Lab Experiments, Lecture Topics, and Student Assignment of the Course

Week	Experiment	Lecture topic	Group work	Student assignment
**1**		Course overview and safety		
**2**	Lab 1. Preparation of metal complexes: cadmium diethyldithiocarbamate (Cd(DDTC)_2_) and zinc diethyldithiocarbamate (Zn(DDTC)_2_)	Metal complexes, sample purification, lab notebook and lab report		Pre lab 1
**3**	Lab 2. Testing stability of metal complexes using thermogravimetry (TGA)	TGA, data processing and graphing		Pre lab 2 & Exp. One report
4	Lab 3: Fluorescence quantum yield measurements using UV–vis absorption and emission spectroscopy	Optical properties of quantum dots (QDs)		Pre lab 3 & Exp. Two report
**5**	Lab 4: Synthesis and optical properties of CdS, and ZnS nanocrystals	Inert atmosphere techniques for the synthesis of inorganic nanocrystals	Y	Pre lab 4 & Exp. Three report
**6**	Lab 5: Synthesis of CdS/ZnS core/shell nanocrystals	Core/shell nanocrystals	Y	Pre lab 5 & Exp. Four report
**7**	Lab 6: Solid state modeling and X-ray diffraction (XRD) for structure characterization	Crystal structures and X-ray diffraction (XRD)		Pre lab 6 & Exp. Five report
**8**	Lab 7: Sensitized solar cells	Applications of inorganic materials and solar cells	Y	Pre lab 7 & Exp. Six report
**9**	Research project	Literature search and project report		Exp. Seven report
**10**	Research project			Project title and abstract
**11**	Research project	How to give a good presentation?		3–5 research papers
**12**	Research project	One-to-one meeting for individual project		
**13**	Final presentations			Final written report

### Part 1 of the Course (First 8 Weeks)

**Scheme 1:**
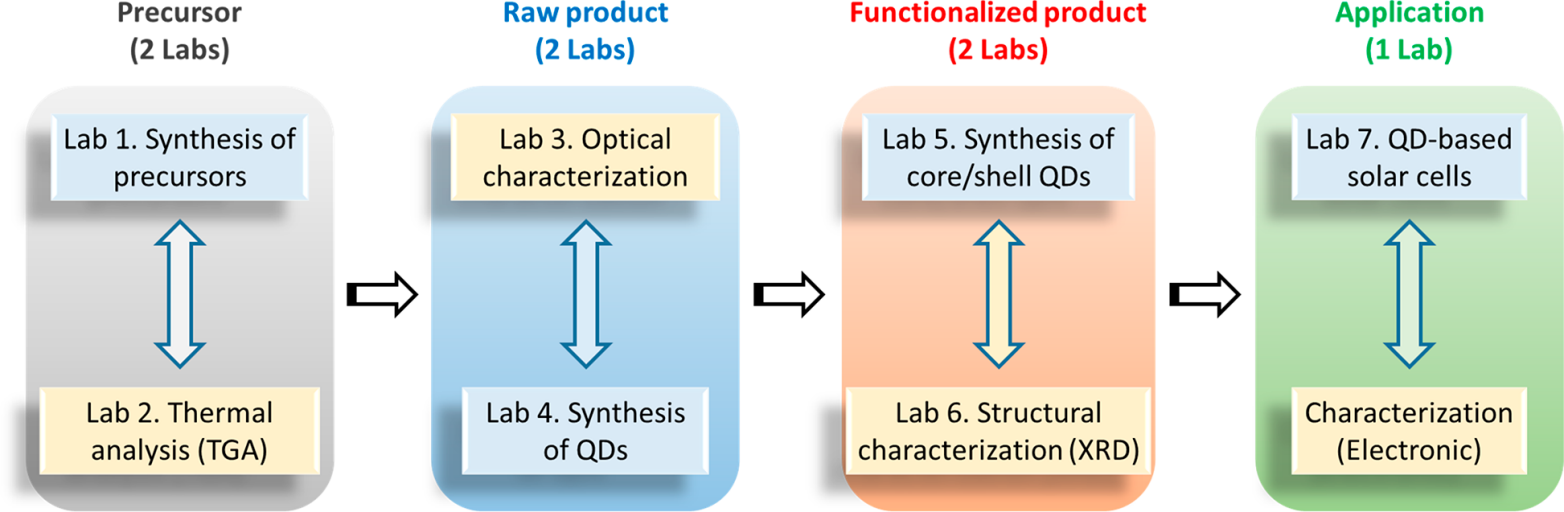
Design of the Scaffolded, Inquiry-Based Portion of the *Inorganic Chemistry Laboratory Technique* course

In the first part of the course, the first 8 weeks, students work through laboratories 1–7 on the synthesis of inorganic metal complexes and nanoparticles, the characterization techniques used to study their structural and optical properties, and green energy application of inorganic materials (Table 1). To enhance students’ learning and help them understand the full picture of the field of inorganic chemistry, we specifically designed these laboratories to be scaffolded and inquiry-based. The laboratories all connect to, and build upon, each other under the broad theme of inorganic materials where students must analyze their results, form conclusions, and then develop communication plans on their own (see Course Assignments section for more details). The series of syntheses and characterization experiments build upon and complement each other over these first 8 weeks (Scheme 1; Note: Laboratories focused on synthesis (1, 4, 5, and 7) and characterization (2, 3, and 6) are shown in pale blue and pale yellow, respectively, in the scheme). The scaffolded design demonstrates to the students the interconnectedness of different areas of inorganic chemistry, which helps students to have a better understanding of the aim of each lab and how to apply each lab technique to a bigger research project.

**Scheme 2:**
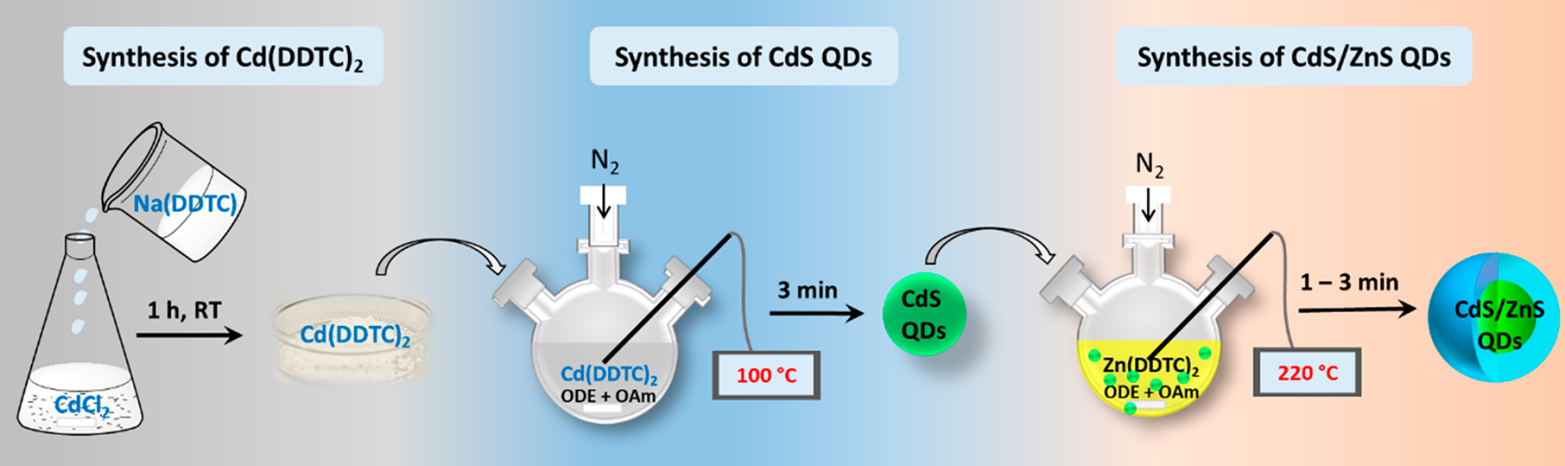
Schematic of the Synthetic Methods Students Used in Part 1 of the Course

On the first day, the course starts with a broad introduction of the class, including the format, content, and requirements of the course. For the first two lab experiments, the students work individually on the synthesis (Lab 1, Scheme 1) and thermal stability of metal complexes cadmium diethyldithiocarbamate (Cd(DDTC)_2_) and zinc diethyldithiocarbamate (Zn(DDTC)_2_) by thermogravimetry (TGA) analysis (Lab 2). The metal complex Cd(DDTC)_2_ is then used to synthesize luminescent cadmium sulfide (CdS) quantum dots (QDs) through a thermal decomposition and growth method in the third and fourth laboratories.^25,26^ UV–vis absorption and emission spectroscopies are utilized to study the optical properties of the QDs (Lab 3). In addition, other inorganic materials such as cesium lead bromide (CsPbBr_3_) perovskite nanocrystals are also provided in the optical measurements in Lab 3, to show students the broad variety of optical properties of inorganic materials including absorption and emission wavelengths, emission quantum efficiencies, *etc*. Laboratories 5 and 6 explore functional CdS/ZnS core/shell QDs for enhanced optical properties and stability. The core/shell QDs are synthesized by surface passivation of CdS QDs with ZnS shell using Zn(DDTC)_2_ as a shell precursor (Scheme 2).^27,28^ The crystal structure of the inorganic CdS core and CdS/ZnS core/shell QDs is studied by powder X-ray diffraction (XRD, lab 6). We also designed one lab experiment titled “Sensitized solar cells” (Lab 7) focused on the application of inorganic nanomaterials. This lab experiment was designed to help students understand the importance of functional inorganic materials and enhance their problem-solving skills by connecting the laboratory experiment with real world applications in green energy harvesting. For three experiments on the synthesis of CdS QDs (Lab 4), functional CdS/ZnS core/shell QDs (Lab 5), and the application of inorganic materials (Lab 7), the students work in pairs (Table 1). A more detailed description of Experiment 5, as an example of how these laboratories are run, and the syllabus of the course are included in the Supporting Information.

### Part 2 of the Course (Last 4 Weeks)

Project-based learning (PBL) is implemented in Part 2 of the course. The goal of this PBL module, covering weeks 9–13, is for students to apply the lab techniques they learned while developing their skills in finding background literature, identifying research questions, and designing an experimental plan. The “project” students work toward is a small research proposal in the field of inorganic materials. Students have the opportunity to pick one topic in inorganic materials that they are interested in and would like to focus on for their proposal. We hope that by varying the styles of the laboratories (inquiry-based lab experiments and laboratories focused on proposal development) and exposing them to as many different ways of learning as possible, we will spark students’ imagination in chemistry research.

Intrinsic motivation, or engaging in activities for the inherent rewards of the behavior itself, is critical for the learning process based on self-determination theory.^21^ In the second part of the course, we encourage students to connect the project to their own career goals and/or interests. Therefore, a very broad range of topics are allowed for the proposal. These topics could be anything related to the development of functional inorganic materials and applications, including the synthesis and applications of inorganic metal complexes, surface modification of semiconductor nanocrystals for enhanced properties and water-soluble nanomaterials, photostability of semiconductor nanocrystals for nanomaterial-based solar cells and photocatalysis in green energy applications, and more. Each student is asked to find 3 to 5 recent research papers (ideally within the past 10 years), identify a fundamental research question(s), and develop their own proposal. While it is not feasible for students to test their proposed experiments as part of this course due to limitations in time and required instruments and chemicals, the students are still practicing important and authentic research skills by working on this proposal. Specifically, students need to justify the rationale of the experimental design, formulate their research hypothesis and expected results, and identify potential challenges in the projects. This project requires some independent literature research, and the related experiments might go beyond what the students learned from the series of experiments in part 1 of the course. Therefore, we provide students with sufficient background information, including a specific lecture on “Literature search”, as well as a one-to-one discussion and feedback for their experimental design of the final project.

Throughout both parts of the course, students are given opportunities to work both individually and in groups. For example, three experiments (Laboratories 4, 5, and 7, Table 1) required high temperature synthesis and/or more deliberate lab operations during the semester, so students work in pairs on these experiments. For the rest of the experiments in the first part of the course, students work individually. Additionally, during the PBL module, students work individually. However, even when students were working individually, we incorporated opportunities for peer learning in which students can both give and receive feedback through peer reviews, during the in-class discussions, and during their final presentations.

## Course Assignments

Throughout Part 1 of the course, students are required to complete prelab assignments where they are asked to develop their own hypotheses and answer questions related to the background and expected results of the experiments based on their hypotheses. Students also complete a postlab report for each experiment where they conduct detailed data analysis and discussion including possible experimental errors and if their hypothesis is correct or flawed.

In Part 2 of the course, the project is broken into a few smaller assignments before students turn in their final product. In week 10 of the course, students submit their project titles and abstract. In week 11, students submit the 3–5 research papers they will be using for their proposal. This assignment is intended to make the students more comfortable reading scientific papers. It also reinforces the connections between the various synthetic and physical experiments performed during the semester. The final product of this project-based learning module is a written project summary and an oral presentation of the students’ proposed project to their classmates at the end of the semester. The written proposal includes (1) Abstract/Objective of the project on inorganic materials (5%); (2) Background and Significance of the specific field of the work (25%); (3) Experimental Description of the synthetic method and characterization techniques (30%); (4) Properties and Applications of the inorganic productor (25%); (5) Conclusion and Outlook (10%); and (6) References of the report (5%) (Note: sections 3 and 4 could be combined as necessary). These final assignments help students develop professional scientific communication skills.

## Hazards and Safety

The inorganic chemistry laboratory contains many potential hazards. Comprehensive safety rules as well as a few additional safety notes related to COVID-19, such as personal protective equipment, are offered to students on the first day, which is considered a starting point for the safe laboratory practice of the course. Specific safety guidelines on the safe conduct of each experiment are provided to students at the beginning of the laboratory period every week. The hazards and safety information are also listed in the course syllabus and lab manuals.

Care should be taken to minimize exposure to all organic solvents and inorganic chemicals used in the experiments by using appropriate eye protection, gloves, and fume hoods (especially with the high temperature reaction for the synthesis of inorganic nanocrystals). QDs and perovskite nanocrystals are synthesized by using highly toxic heavy metal ions such as cadmium and lead. Students are required to wear nitrile gloves and goggles at all times and maintain extreme care during handling these nanomaterials. The reactions for QD synthesis are conducted under an inert gas and high temperatures. There is a potential burn hazard for using a stirring hot plate and oil bath. Caution should be taken to avoid touching the surface of a hot plate and the hot glass flask during the high temperature synthesis. Care should be taken with the use of the centrifuge to avoid unbalanced centrifuge tubes, which can cause damage and injure the operator and other laboratory personnel. Care should be taken to properly dispose of all liquid waste, sharp needles, and broken or used glass pipets in designated waste containers inside the fume hood during or after the experiments.

## Study Methodology

End-of-semester student evaluations were used to determine broad outcomes for students across the first three semesters that this course was taught. Pilot survey data was collected from the seven students enrolled in this course in Spring 2023 (This study was determined to be exempt by the Syracuse University Institutional Review Board, IRB#: 22-403). Students were asked to complete a survey at both the beginning and end of the semester. The pre/postcourse questions assessed students’ interest in chemistry, science self-efficacy, and science identity (Table 2). The additional postcourse survey questions assessed students’ perceptions of whether specific aspects of the course (i.e., scaffolded, inquiry-based laboratories, and project-based learning) impacted their understanding, interest, and skills, as well as their perceptions of their overall experience in the course (Table 3). Most of the survey questions were pulled from previously published tools and modified to fit the context of this course.^29,30^ All questions used a Likert scale of 1-strongly disagree, 2-disagree, 3-neither agree nor disagree, 4-agree, and 5-strongly agree. Responses to the pre- and postsurvey questions were averaged across the students and compared qualitatively. Due to the small sample size, statistical analyses were not used. For the questions asked only on the postsurvey, we calculated the percentage of students that responded with each of the five Likert-scale options and compared these percentages qualitatively.

**Table 2:** Pre- and Post-course Survey Questions on Students’ Interests, Science Self-Efficacy, Science Identity, and Sense of Belonging

Theme	Number	Survey Questions
Interests in chemistry	1	I am interested in the field of chemistry.
	2	I am interested in the field of inorganic chemistry.
Science self-efficacy	3	I know where I can find resources, including scientific literature, for a research project.
	4	I can generate research questions for a project.
	5	I can analyze and interpret the meaning of data/observations from my lab experiments.
	6	I can create explanations for the results of experiments.
	7	I can solve problems in inorganic chemistry research.
	8	I can think critically about inorganic chemistry research.
	9	Whether the science content is difficult or easy, I am sure that I can understand it.
Science identity	10	I have come to think of myself as a “scientist”.
	11	My interest in science is an important reflection of who I am.

**Table 3:** Post-course Survey Questions on the Scaffolded, Inquiry-Based Lab Experiments (Part 1 of the Course), Project-Based Learning (Part 2 of the Course), and Overall Experience

Theme	Number	Survey Questions
Scaffolded, inquiry-based lab experiments	1	The scaffolded inquiry-based lab experiments in the first part of this course were a good way to learn about the subject matter.
	2	The scaffolded inquiry-based lab experiments in the first part of the course enhanced my problem-solving skills.
	3	The scaffolded inquiry-based lab experiments in the first part of the course enhanced my critical thinking skills.
Project-based learning part of the course	4	The project-based learning part (second part) of this course was a good way to learn about the subject matter.
	5	The project-based learning part of this course enhanced my problem-solving skills.
	6	The project-based learning part of this course enhanced my critical thinking skills.
Overall experience	7	This course motivated me to search for scientific information.
	8	I am more motivated to learn course materials when I see a potential application to society.
	9	I get personal satisfaction when I can combine my chemistry knowledge with applications, such as lighting devices and solar cells in green energy harvesting and sustainability.
	10	Chemistry courses become more interesting for me when they connect with my personal values.

## Results

### Broad Student Outcomes

Given the small enrollment in this course, both the instructor and the TA have been able to provide quality one-on-one support to each student enrolled in the course each semester it has been taught. The new course has had a 100% passing rate, and very positive student evaluations each semester it has been offered. Students indicated a high level of satisfaction with the developed course, especially with the incorporation of scaffolded, inquiry-based lab experiments. One of the students in the spring 2021 course commented that “*It was really such a great class and the fact that most labs built on each other reaffirmed each concept very well!*”. Another student from the same semester commented that “*This class helped in my overall knowledge of chemistry a lot*.” Students like that the lecture and discussion are a part of the lab since they are directly relevant to the lab content and make the experiments easy to follow. Students also like the collaborative team-based learning that we incorporated in laboratories 4 and 5 to explore the synthesis and properties of inorganic materials. One of the students in the spring 2020 course commented that “*I liked the experiments because they were easy to follow and were relevant to the material. I liked that I got to pick my group and collaborate with them*.”. The student also gave very high-quality presentations on topics such as the synthesis, morphology, stability, and optical properties of functional semiconductor nanomaterials (see an example student presentation in the Supporting Information).

We also wanted to ensure that students experienced the reality of undergoing imperfect chemical reactions during inorganic chemistry research. There are many undergraduate and graduate students who understand fundamental chemistry concepts and theories very well. However, occasionally, those “great” students have encountered trouble in their research, with one possible reason being that they seldom realize the big gap between ideal/theoretical results and results in real experiments. Students in their coursework have studied basic theories and concepts under perfect conditions, which does not happen in the real world. Our goal with this course, using inquiry-based lab design where students must determine the results, analysis, and conclusions and then communicate plans on their own, was to provide students with the opportunity to develop their critical thinking and problem-solving skills. Specifically, the lab experiments emphasize the real conditions and outcomes of the chemical experiments. We let students know the deviation of the ideal results are not always experimental errors, and in reality, experiments never work out perfectly even without human error. For example, solvent partitioning in a separatory funnel is routinely used in the chemistry laboratory as one of the basic purification methods, which requires two solvents that are not miscible with each other and form two layers when mixed together. Usually, one of the solvents is polar, such as water, and the other solvent is nonpolar, such as toluene. We usually expect a compound to dissolve in one solvent rather than another because of the different solubilities in two different solvents based on the concept of “like dissolves like”. However, we are seldom rewarded with perfection. This is not because of “human error” but because of the nature of equilibrium that governs how much of the compound goes in one layer and how much goes in the other. In addition, usually when we do an extraction, we like to see a good separation between two clear layers. One practical problem when we clean the inorganic nanoparticles using “solvent extraction” after QD synthesis in laboratories 4–5 is forming a cloudy “solution” instead of two well-separated layers. The nanoparticles could slowly precipitate out from the mixture of polar/nonpolar solvents. However, through the in-class discussion comparing the different sample purification techniques available in the lab, students can come up with a solution to this issue by centrifugation to separate the nanocrystals from solution. Ultimately, by highlighting the imperfect reality of inorganic chemistry experiments, we hope to better prepare students to solve these types of problems in their future work.

### Survey Results

**Figure 1:**
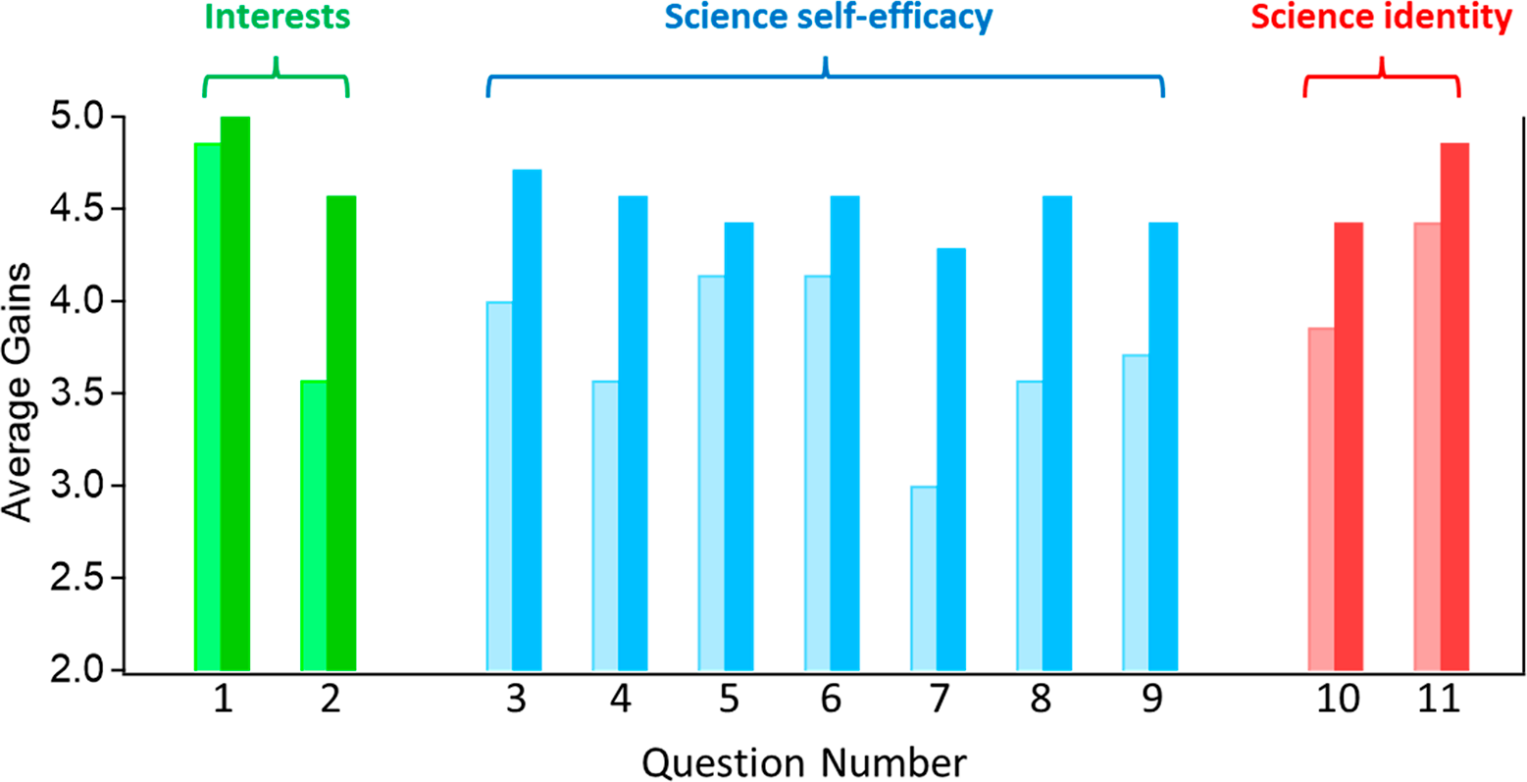
Comparison of student response from the pre- and postcourse survey questions as indicated by the lighter and darker bars, respectively.

We additionally collected pre/postcourse survey data for questions about students’ interests, science self-efficacy, and science identity (Table 2). Although we only have data from seven students in Spring 2023 and were unable to analyze these data statistically, we did find some interesting trends in their survey responses (Figure 1). First, we found that while these students did not have a notable increase in their interest in Chemistry broadly (M_pre_ = 4.9, M_post_ = 5.0, Question 1 in Figure 1; M = average), they did have a notable increase in their interest in the field of Inorganic Chemistry (M_pre_ = 3.6, M_post_ = 4.6, Question 2 in Figure 1). It is important to note here that these students started with a much higher interest in Chemistry (strongly agree on average) compared to that in Inorganic Chemistry (between neutral and agree on average), which makes sense given that these students are juniors and seniors majoring in Chemistry. However, it is exciting to see the increase in their interest specifically toward Inorganic Chemistry after taking the lab course (M_post_ = 4.6, between agree and strongly agree on the postsurvey).

We also found notable increases in most of the questions regarding students’ science self-efficacy. These students reported an increase, on average, in their ability to find resources for a research project (M_pre_ = 4.0, M_post_ = 4.7, Question 3 in Figure 1), generate research questions for a project (M_pre_ = 3.6, M_post_ = 4.6, Question 4 in Figure 1), solve problems in inorganic chemistry research (M_pre_ = 3.0, M_post_ = 4.3, Question 7 in Figure 1), think critically about inorganic chemistry research (M_pre_ = 3.6, M_post_ = 4.6, Question 8 in Figure 1), and generally understand the science content (M_pre_ = 3.7, M_post_ = 4.4, Question 9 in Figure 1). Students also reported a slight increase in whether they think of themselves as a “scientist” (M_pre_ = 3.9, M_post_ = 4.4, Question 10 in Figure 1) and whether their interest in science is an important reflection of who they are (M_pre_ = 4.4, M_post_ = 4.9, Question 11 in Figure 1).

As a whole, these pre/postcourse survey results suggest that the course helped improve students’ interest, self-efficacy, and science identity. We recognize that we only have data for seven students, and therefore the findings should be taken with a grain of salt, but these results suggest positive trends that we can further investigate in the future.

**Figure 2:**
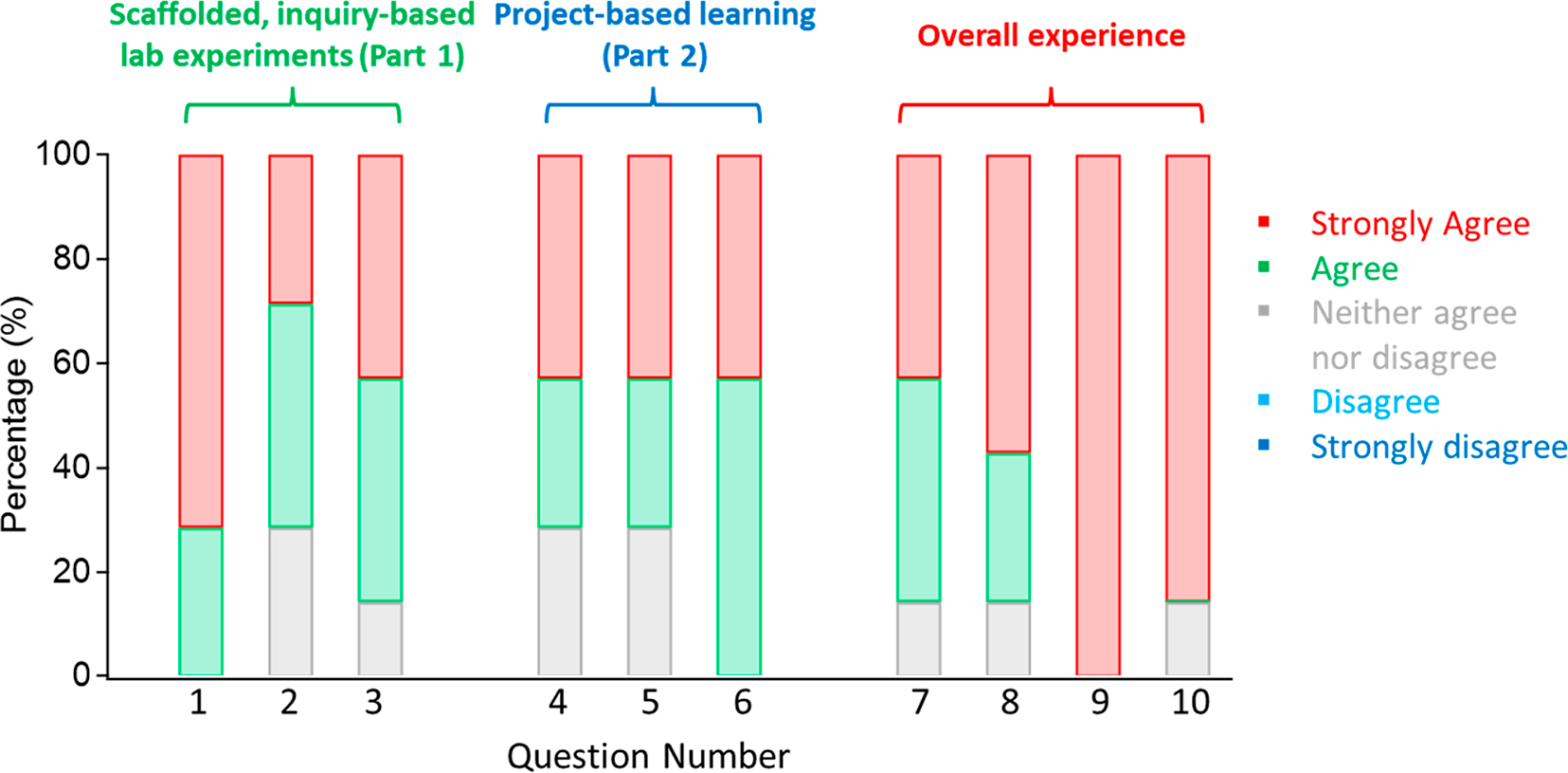
Student response frequencies for the impact of the scaffolded, inquiry-based lab experiments (part 1 of the course), project-based learning (part 2 of the course), and overall experience.

Finally, we asked students to report on their experiences of different aspects of the course on the postsurvey (Table 3). Overall, these students reported very positive experiences of the course (Figure 2); in regard to the scaffolded, inquiry-based portion of the course, all students either agreed or strongly agreed that the scaffolded, inquiry-based lab experiments were a good way to learn about the subject matter (Question 1 in Figure 2). Five of the seven students either agreed or strongly agreed that these lab experiments enhanced their problem-solving skills (question 2 in Figure 2), and six of the seven students either agreed or strongly agreed that these experiments enhanced their critical thinking skills (question 3 in Figure 2). For the PBL portion of the course (questions 4–6 in Figure 2), five of the seven students either agreed or strongly agreed that the PBL portion was a good way to learn about the subject matter and that the PBL portion enhanced their problem-solving skills. All students either agreed or strongly agreed that the PBL portion enhanced their critical thinking skills. Overall, these results suggest that the two portions of the course helped improve these students’ problem-solving and critical thinking skills.

For students’ overall experiences in the course (questions 7–10 in Figure 2), six of the seven students either agreed or strongly agreed that the course motivated them to search for scientific information and that they are more motivated to learn course materials when they see a potential application to society. All seven students strongly agreed that they get personal satisfaction when they can combine their chemistry knowledge with applications, and six of the seven students strongly agreed that chemistry courses become more interesting when they connect with their personal values. These results suggest that providing students with autonomy and opportunities to connect the content with real-world applications, as we did in our course, could lead to more positive experiences for students. However, further investigation and confirmation of these trends should be done due to our small sample size and associated lack of statistical analyses.

## Conclusions

A new inorganic chemistry laboratory technique course was developed to provide students with access to a variety of experimental techniques for exploring new properties and applications of inorganic compounds and nanocrystals. The course incorporated both scaffolded, inquiry-based experiments and project-based learning to give students a more realistic and interesting experience of inorganic chemistry. We also incorporated opportunities for both individual and team-based learning as well as peer review. We hope that the lab experience provided by this course will inspire students to pursue a career in chemistry. While the experiments developed for this course focus on the synthesis and characterization of inorganic materials, the general design of the course (scaffolded, inquiry-based laboratories and the project-based learning module) could be used in other laboratory courses with different topics and lengths.

## Supporting Information Available

Syllabus of the course (PDF)
Lab manual of Experiment 5 including prelab questions as an example of how these laboratories are run (PDF)
Lecture slides for Experiment 5 including postlab questions (PDF)
An example student presentation of the proposal from the PBL module (PDF)
