# Supplementary material for: An Inorganic Chemistry Laboratory Technique Course using Scaffolded, Inquiry-Based Laboratories and Project-Based Learning
Source: J Chem Educ. 2023 Aug 15;100(9):3500–8. doi: 10.1021/acs.jchemed.3c00547 (PMC10501116; doi:10.1021/acs.jchemed.3c00547)
Supplement: Supplementary file 1 — ed3c00547_si_001.pdf [file ed3c00547_si_001.pdf]

Supporting Information for

An Inorganic Chemistry Laboratory Technique Course using Scaffolded, Inquiry-Based Labs and Project-Based Learning

Chun Chu,[a] Jessica L Dewey,[b] Weiwei Zheng *[a]

[a] Department of Chemistry, Syracuse University, Syracuse, New York 13244, United States

[b] Duke Learning Innovation, Duke University, Durham, North Carolina 27708, United States

Corresponding Author

*E-mail: wzhen104@syr.edu

CHE 422: Inorganic Laboratory Technique

Spring 2023, Room 101 Life Science Building
M001: Wednesday 2:15 – 5:15 PM; M002: Tuesday 2 – 5 PM

Instructor: Dr. Weiwei Zheng
Office: Room 3-050, CST
Email: wzhen104@syr.edu
Phone: 315-443-3062
Office hours: by appointment

Course Description:

This class will explore skills in the synthesis and physical characterization of metal complexes and inorganic nanocrystals. We will introduce synthetic methods, crystal structures, characterization techniques, physical properties, and applications of inorganic solids as well as nanomaterials. A separate lecture that presents the concepts of the laboratory experiment(s) will precede the laboratory period(s) devoted to each experiment.

Course goals:

This course intends to help you grow as a chemist by reinforcing the techniques and skills that a synthetic inorganic chemist would employ in the workplace and guiding you towards the development of better reasoning and critical thinking skills for problem solving. We will gain valuable experience on the synthesis and characterization of a variety of inorganic and organometallic compounds.

1. We will learn synthetic methods for a variety of organometallic compounds and inorganic nanocrystals.
2. We will utilize proper physical techniques to characterize solids, including UV-Visible spectroscopy, emission spectroscopy, thermogravimetry (TGA), X-ray powder diffraction (XRD), and some simple sample purification methods such as centrifugation.
3. We will understand and analyze structural and optical properties of inorganic nanomaterials.
4. We will utilize literature for research in the synthesis and properties of inorganic nanomaterials.

Topics Covered:

- Safety in the Laboratory.
- Inorganic Syntheses and Reactions; Inert Atmosphere Techniques.
- Physical Methods for materials characterization: electronic spectroscopy (UV-vis absorption and emission spectroscopy), thermogravimetric analysis (TGA), and X-ray diffraction (XRD).

Materials and textbooks (optional) that are relevant to the course:

- Laboratory Manuals/Handouts will be provided to the students on Blackboard by the Friday before each lab. Safety glasses will be provided.
- Inorganic Chemistry, 5th edition, Shriver & Atkins, Freeman Press, 2010. (*Any inorganic text will be appropriate to use as a reference)
- Solid State Chemistry: An Introduction, 4th Edition. Smart, Lesley E. CRC Press, 2012

Undergraduate Laboratory Supervisor: Gary Bonomo, LSC 012A, email: gbonomo@syr.edu
Teaching Assistants: Chun Chu, email: cchu06@syr.edu

Lab Safety

- First and foremost you will be required to wear safety goggles at all times when in the laboratory. If you are caught without safety glasses on more than one occasion you will be asked to leave the lab.
- Shorts and open-toed shoes are not permitted in the laboratory.
- No food or drink is allowed in the laboratory at any time.
- Do not sniff or taste any of the chemicals you will be using.
- Toxic substances must be used only under the hood. You will be responsible for looking up and understanding the Material Safety Data Sheet (MSDS) of all chemicals used in the laboratory.
- All cell phones, pagers, CD players, MP3 players, etc. must be turned off (or at the very least on silent) while in the laboratory.

Grading policy:

A-C scale, with 92-100 A; 89-91 A-; 86-88 B+; 82-85 B; 79-81 B-; 76-78 C+; 72-75 C; <72 C-

Prelab Questions (10%), Notebook and Laboratory Work (20%), Lab Report (30%), Post lab questions (10%). Final Project (30%) including final Written Report (15%) and Final Presentation (15%). Attendance: 2%.

Final project and presentation:

The final project consists of two components, a written report and an oral presentation (~15 min in class, including slides of Introduction, approach, results, conclusion, and references); each component contributes 15% to the overall course grade, for a total contribution of 30%.

Attendance and Tardiness: In order to be successful in this course, your attendance in each lab is mandatory. There will be no makeup labs offered for this course. If you are more than 15 minutes late for class, you will not be permitted to perform the lab.

Notebook and Lab Report: Please buy a regular notebook for this course. No need to buy carbon copy lab notebooks. You do not need to buy a textbook for this class. I will provide handouts or post the relevant material on Blackboard for you. You should print out each lab and must complete a summary of the lab procedure in your notebook before you come to class.

Each lab report is due at the beginning of the lab in the following week. No late assignments will be accepted under any circumstances; emergency situations will be dealt on a case-by-case basis.

Note: We are bound by the rules, regulations, and the honor code of our university in all matters related to this course: attendance, registration, and examinations.

Tentative course outline:

Week	Date	Experiment/Lecture Topic	Due
1	1/18	*No lab in the first week*	
2	1/25	**Course overview, safety and polices**	
3	2/1	**Experiment 1**: Preparation of metal complexes: cadmium diethyldithiocarbamate ($Cd(DDTC)_2$) and zinc diethyldithiocarbamate($Zn(DDTC)_2$); Lab notebook/report, and Sample purification.	Pre lab 1
4	2/8	**Experiment 2**: Testing stability of metal complexes using thermogravimetry (TGA); Data processing and graphing.	Pre lab 2 & Exp. 1 report
5	2/15	**Experiment 3**: Fluorescence quantum yield measurements using UV-vis absorption and emission spectroscopy; Optical properties of quantum dots (QDs).	Pre lab 3 & Exp. 2 report
6	2/22	**Experiment 4**: Synthesis and optical properties of CdS, ZnS, and CdZnS nanocrystals; Inert atmosphere techniques for the synthesis of inorganic nanocrystals.	Pre lab 4 & Exp. 3 report
7	3/1	**Experiment 5**: Synthesis of CdS/ZnS core/shell nanocrystals using $Zn(DDTC)_2$ as ZnS shell precursor; Core/shell nanocrystals.	Pre lab 5 & Exp. 4 report
8	3/8	**Experiment 6**: Solid state modeling and X-ray diffraction (XRD) for structure characterization of nanocrystals; Crystal structures and XRD.	Pre lab 6 & Exp. 5 report
9	3/15	*Spring Break (no classes)*	
10	3/22	**Experiment 7**: Sensitized solar cells; Solar cells.	Pre lab 7 & Exp. 6 report
11	3/29	**Final project**: composition and size-dependent optical and surface properties of nanocrystals. Literature searching and final project report.	Exp. 7 report
12	4/5	**Final project**	Project title and abstract
13	4/12	**Final project**	3-5 research papers
14	4/19	**Final project**: Lecture on "How to give a good presentation?" followed by one-to-one meetings.	
15	4/26	**Final presentations**	Final report

Stay Safe Pledge

Syracuse University's Stay Safe Pledge reflects the high value that we, as a university community, place on the well-being of our community members. This pledge defines norms for behavior that will promote community health and wellbeing. Classroom expectations include the following: wearing a mask that covers the nose and mouth as needed, maintaining a reasonable distance from others, and staying away from class if you feel unwell. Repeated violations will be treated as violations of the Code of Student Conduct and may result in disciplinary action.

Student Health

Mental health and overall well-being are significant predictors of academic success. As such it is essential that during your college experience you develop the skills and resources effectively to navigate stress, anxiety, depression and other mental health concerns. Please familiarize yourself with the range of resources the Barnes Center provides (ese.syr.edu/bewell) and seek out support for mental health concerns as needed. Counseling services are available 24/7, 365 days a year, at 315.443.8000.

Use of Class Materials and Recordings

Original class materials (handouts, assignments, tests, etc.) and recordings of class sessions are the intellectual property of the course instructor. You may download these materials for your use in this class. However, you may not provide these materials to other parties (e.g., web sites, social media, other students) without permission. Doing so is a violation of intellectual property law and of the student code of conduct.

University Attendance Policy

Attendance in classes is expected in all courses at Syracuse University. Students are expected to arrive on campus in time to attend the first meeting of all classes for which they are registered. Students who do not attend classes starting with the first scheduled meeting may be academically withdrawn as not making progress toward degree by failure to attend. Instructors set course-specific policies for absences from scheduled class meetings in their syllabi.

It is a federal requirement that students who do not attend or cease to attend a class to be reported at the time of determination by the faculty. Faculty should use "ESPR" and "MSPR" in Orange Success to alert the Office of the Registrar and the Office of Financial Aid. A grade of NA is posted to any student for whom the Never Attended flag is raised in Orange SUccess. More information regarding Orange SUccess can be found at http://orangesuccess.syr.edu/getting-started-2/.

Students should also review the University's religious observance policy and make the required arrangements at the beginning of each semester.

Religious Observances Notification and Policy

Syracuse University's Religious Observances Policy (https://policies.syr.edu/policies/university-governance-ethics-integrity-and-legal-compliance/religious-observances-policy/) recognizes the diversity of faiths represented in the campus community and protects the rights of students, faculty, and staff to observe religious holy days according to their traditions. Under the policy, students are given an opportunity to make up any examination, study, or work requirements that may be missed due to a religious observance, provided they notify their instructors no later than the academic drop deadline. For observances occurring before the drop deadline, notification is required at least two academic days in advance. Students may enter their observances in MySlice under Student Services/Enrollment/My Religious Observances/Add a Notification.

Orange Success

(tools to access a variety of SU resources, including ways to communicate with advisors and faculty members) can be found here, at: http://orangesuccess.syr.edu/getting-started-2/

Diversity and Disability

Syracuse University values diversity and inclusion; we are committed to a climate of mutual respect and full participation. There may be aspects of the instruction or design of this course that result in barriers to your inclusion and full participation in this course. I invite any student to contact me to discuss strategies and/or accommodations (academic adjustments) that may be essential to your success and to collaborate with the Center for Disability Resources (CDR) in this process.

If you would like to discuss disability-accommodations or register with CDR, please visit Center for Disability Resources at https://disabilityresources.syr.edu. Please call (315) 443-4498 or email disabilityresources@syr.edu for more detailed information. The CDR is responsible for coordinating disability-related academic accommodations and will work with the student to develop an access plan. Since academic accommodations may require early planning and generally are not provided retroactively, please contact CDR as soon as possible to begin this process.

Academic Integrity Policy

Syracuse University's Academic Integrity Policy reflects the high value that we, as a university community, place on honesty in academic work. The policy defines our expectations for academic honesty and holds students accountable for the integrity of all work they submit. Students should understand that it is their responsibility to learn about course-specific expectations, as well as about university-wide academic integrity expectations. The policy governs appropriate citation and use of sources, the integrity of work submitted in exams and assignments, and the veracity of signatures on attendance sheets and other verification of participation in class activities. The policy also prohibits students from submitting the same work in more than one class without receiving written authorization in advance from both instructors. Under the policy, students found in violation are subject to grade sanctions determined by the course instructor and non-grade sanctions determined by the School or College where the course is offered as described in the Violation and Sanction Classification Rubric. Syracuse University students are required to read an online summary of the University's academic integrity expectations and provide an electronic signature agreeing to abide by them twice a year during pre-term check- in on MySlice.

Discrimination or Harassment

Federal and state law, and University policy prohibit discrimination and harassment based on sex or gender (including sexual harassment, sexual assault, domestic/dating violence, stalking, sexual exploitation, and retaliation). If a student has been harassed or assaulted, they can obtain confidential counseling support, 24-hours a day, 7 days a week, from the Sexual and Relationship Violence Response Team at the Counseling Center (315-443-8000, Barnes Center at The Arch, 150 Sims Drive, Syracuse, New York 13244). Incidents of sexual violence or harassment can be reported non-confidentially to the University's Title IX Officer (Sheila Johnson Willis, 315-443-0211, titleix@syr.edu, 005 Steele Hall). Reports to law enforcement can be made to the University's Department of Public Safety (315-443-2224, 005 Sims Hall), the Syracuse Police Department (511 South State Street, Syracuse, New York, 911 in case of emergency or 315-435-3016 to speak with the Abused Persons Unit), or the State Police (844-845-7269). I will seek to keep information you share with me private to the greatest extent possible, but as a professor I have mandatory reporting responsibilities to share information regarding sexual misconduct, harassment, and crimes I learn about with the University's Title IX Officer to help make our campus a safer place for all.
