# Supplementary material for: An Inorganic Chemistry Laboratory Technique Course using Scaffolded, Inquiry-Based Laboratories and Project-Based Learning
Source: J Chem Educ. 2023 Aug 15;100(9):3500–8. doi: 10.1021/acs.jchemed.3c00547 (PMC10501116; doi:10.1021/acs.jchemed.3c00547)
Supplement: Supplementary file 2 — ed3c00547_si_002.pdf [file ed3c00547_si_002.pdf]

Supporting Information for

An Inorganic Chemistry Laboratory Technique Course using Scaffolded, Inquiry-Based Labs and Project-Based Learning

Chun Chu,[a] Jessica L Dewey,[b] Weiwei Zheng *[a]

[a] Department of Chemistry, Syracuse University, Syracuse, New York 13244, United States

[b] Duke Learning Innovation, Duke University, Durham, North Carolina 27708, United States

Corresponding Author

*E-mail: wzhen104@syr.edu

Experiment 5. Synthesis of CdS/ZnS core/shell quantum dots

Introduction:

Colloidal semiconductor nanocrystals (NCs), also termed ‘‘quantum dots’’ (QDs), are composed of an inorganic core, made up of between a few hundred and a few thousand atoms, surrounded by an organic outer layer of surfactant molecules (ligands). Their small size results in an observable quantum confinement effect, defined by an increasing bandgap accompanied by the quantization of the energy levels to discrete values. The size-dependent optical properties of NCs have been the focus of significant research over the past two decades.

The nanometric crystal size also results in a very high surface-to-volume ratio. The coordination sphere of this high population of surface atoms partially occurs via complex formation with the stabilizing ligands. Nevertheless, a significant fraction of these organically passivated core NCs typically exhibit surface related trap states acting as fast non-radiative de-excitation channels for photo-generated charge carriers, thereby reducing the fluorescence quantum yield (QY). An important strategy to improve NCs' surface passivation is their overgrowth with a shell of a second semiconductor, resulting in core/shell (CS) systems. In this manner, the fluorescence efficiency and stability against photo-oxidation of various types of semiconductor NCs has seen significant improvement. Furthermore, by the appropriate choice of the core and shell materials, it is possible to tune the emission wavelength in a larger spectral window than with both materials alone.

Colloidal core/shell nanocrystals contain at least two semiconductor materials in an onion-like structure. The core and the shell are typically composed of type II–VI, IV–VI, and III–V semiconductors, with configurations such as CdS/ZnS, CdSe/ZnS, CdSe/CdS, and InAs/CdSe core/shell nanocrystals.

The possibility to tune the basic optical properties of the core nanocrystals, for example, their fluorescence wavelength, quantum yield, and lifetime, by growing an epitaxial-type shell of another semiconductor has fueled significant progress on the chemical synthesis of these systems. In such core/shell nanocrystals, the shell provides a physical barrier between the optically active core and the surrounding medium, thus making the nanocrystals less sensitive to environmental changes, surface chemistry, and photo-oxidation. The shell further provides an efficient passivation of the surface trap states, giving rise to a strongly enhanced fluorescence quantum yield. This effect is a fundamental prerequisite for the use of nanocrystals in applications such as biological labeling and light-emitting devices, which rely on their emission properties. Organically passivated QDs have low fluorescence quantum yield due to surface related trap states. Core/shell NCs address this problem because the shell increases quantum yield by passivating the surface trap states. In addition, the shell provides protection against environmental changes, photo-oxidative degradation, and provides another route for modularity. Precise control of the size, shape, and composition of both the core and the shell enable the emission wavelength to be tuned over a wider range of wavelengths than with either individual semiconductor. These materials have found applications in biological systems and optics.

Classification of core–shell semiconductor nanocrystals. Core/shell semiconductor nanocrystal properties are based on the relative conduction and valence band edge alignment of the core and the shell. In type I semiconductor heterostructures, the electron and holes tend to localize within the core. In type II heterostructures, one carrier is localized in the shell while the other is localized in the core.

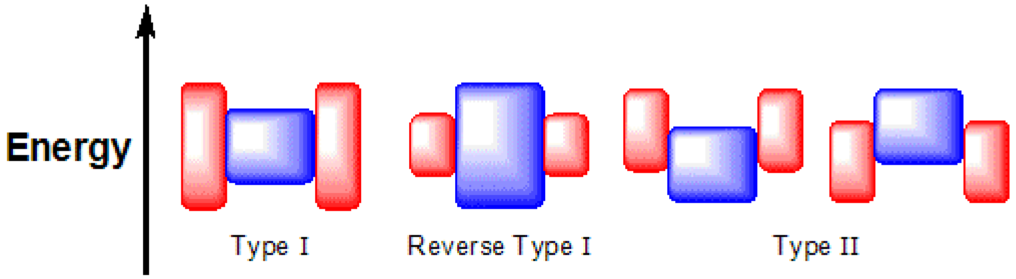

Figure S1: The three types of core/shell nanocrystals. The upper and lower edges represent the upper and lower energy edges of the core (blue) and the shell (red).[4]

Type I: In a Type I core/shell NC, the bandgap of the core is smaller than that of the shell. Both the conduction and valence band edges of the core lie within the bandgap of the shell, which confines both electrons and holes in the core. This can be seen in figure S1, where the electron and hole of an exciton at the CdSe (bandgap:1.74 eV)/CdS (bandgap:2.42 eV) interface occupy energy states within the CdSe core, which corresponds to the lowest available energy separation. The emission wavelength due to radiative electron-hole recombination within the core is slightly redshifted compared to uncoated CdSe.

Reverse Type I: In the reverse type I configuration, the core has a wider bandgap than the shell, and the conduction and valence band edges of the shell lie within those of the core. The lowest available exciton energy separation occurs when the charge carriers are localized in the shell. Changing the shell thickness tunes the emission wavelength.

Type II: In the type II configuration, the valence and conduction band edge of the core are both lower or higher than the band edges of the shell. An example of a type II is shown in figure S1, ZnTe (bandgap:2.26) /CdSe (bandgap:1.74). The lowest energy separation of the electron and the hole will occur when the hole is confined in the ZnTe core valence band and the electron is confined in the CdSe shell conduction band. The emission wavelength will be determined by the energy difference between these occupied states, as shown by the red arrow, which will be at a lower energy than either of the individual bandgaps. The emission wavelength can be significantly red shifted compared to the unpassivated core.

Objectives:

In this lab, we will synthesis ZnS shell passivated CdS QDs (i.e. CdS/ZnS core/shell QDs) and study their optical properties. We will measure the absorption and emission (fluorescence) spectra of the samples using an UV-vis spectrophotometer and an emission spectrometer, respectively.

Chemicals:

1. CdS quantum dots (QDs)
2. zinc diethyldithiocarbamate ($Zn(DDTC)_2$)
3. 1-octodecene (ODE)
4. Oleylamine (OAm)
5. Toluene
6. Acetone or Ethanol
7. Mineral oil

Materials:

1. (1) 25 mL 3-neck round bottom flask
2. (3) rubber septum
3. (1) magnetic stir bar
4. (1) 1 mL plastic syringe and needle
5. (1) 5 mL plastic syringe and needle
6. (1) 5 mL glass syringe
7. (3) 15 mL centrifuge tube
8. (1) Glass Petri Dish (for oil bath)
9. (1) Digital probe thermometer
10. (1) Timer
11. Disposable glass pipettes
12. quartz cuvette

Instruments:

Hot plate
Cary Series UV-Vis Spectrophotometer
Cary Eclipse Fluorescence Spectrophotometer

Procedure:

Part A: Single Source Precursor Shelling of ZnS on the Surface of CdS Quantum Dots

1. Place one magnetic stir bar in the 25 mL 3-neck round bottom flask.
2. Weigh out 0.368 g of the $Zn(DDTC)_2$ using an analytical balance and add it to the round bottom flask.
3. Get synthesized CdS core nanoparticles in a 15 mL centrifuge tube from your TA (~1/2 of the CdS QDs from a synthesis reaction in Experiment 4). Using the 5 mL plastic syringe, measure out 4.5 mL of ODE and add it to the centrifuge tube. Sonicate the solution and then transfer them into the 25 mL 3-neck round bottom flask.
4. Using the 3 mL plastic syringe, measure out 1.5 mL of OAm and add it to the previous centrifuge tube. Sonicate the solution and then transfer the solution into the 25 mL 3-neck round bottom flask.
5. Cover each of the openings on the 3-neck round bottom flask with rubber septums and stir the solution on a hotplate until the mixture is homogeneous.
6. Vacuum the system by inserting a needle into the rubber septum (middle one of the flask) that connects the double manifold for 30 minutes. Once the vacuuming time is finished use the double manifold to fill the round bottom with N_2 gas. (TA will help on this step)
7. Place the round bottom into the oil bath, on a hot plate, and puncture one of the septums with the thermocouple and allow it to sit in the solution in a place that it will not interact with the stir bar (use a needle to puncture the septum first in order to make a hole large enough for the thermocouple to fit through).

8. Using a new needle, puncture another septum and place a drop of OAm into the tip of the needle.
9. Label the three centrifuge tubes (0 minutes, 1 minute, and 3 minutes) and place 1 mL of toluene into each tube.
10. Set the stir rate to 800 – 900 RPM and set the temperature of the hotplate to 500 °C. Monitor the temperature of the reaction by the thermometer and make sure the temperature of the reaction reaches to 220 °C, then turn off the heat of the hotplate. (Note: The temperature should be able to maintain ~220 °C with the 3 min reaction period after the heat was turned off.)
11. Use the 5 mL glass syringe to withdraw 1.5 mL of the reaction solution at each of the reaction times (0 minutes, 1 minute, and 3 minutes) and inject into their corresponding centrifuge tubes.
12. Add ~3 mL of ethanol into each of the centrifuge tubes to crash the quantum dots out of solution.
13. Centrifuge each of the solutions for 5 minutes at 5000 RPM.
14. Discard the supernatant then add in an additional 1 mL of toluene and sonicate to reaction to dissolve the quantum dot pellet into solution.
15. Centrifuge the solutions one additional time to remove any byproducts. The quantum dots will remain in solution.
16. Properly clean up your work area and glassware. Use a magnetic stir bar retriever to take the stir bar out of the 3-neck flask and put it into the beaker which is used to store stir bars. Then, clean up your glassware using soap water and rinse it with DI water. Following the guide in "**Cleaning and waste disposal**" (Page 5).

Part B. Measuring the UV-Vis Spectra

1. Clean the quartz cuvette with toluene and dry, using a kimwipe, before each scan. The solvent we will be using for our scans is toluene.
2. Turn on the UV-Vis and allow the instrument to boot up.
3. Take a baseline scan of the solvent, and "zero" the instrument.
4. Add in two drops of your concentrated quantum dot solution into the toluene filled cuvette using a glass pipet and make sure the mixture is homogeneous by mixing well with the pipet.
5. Run the UV-Vis scan and save the file as a CSV format.
6. Remove the cuvette from the UV-Vis and dispose of the sample in the organic waste container. No chemicals can be disposed in the sink.
7. Repeat steps 4 – 6 with all of the quantum dot samples.
8. Clean up your space and the cuvette and report the observations.

Part C: Measuring the Fluorescence Spectra

TA will turn on the instrument for about 30 min to warm up the instrument prior to the emission measurement. You will follow similar steps as above. Settings for the fluorescence instrument will be introduced by your TA in the lab.

Safety notes:

Quantum dots are synthesized using highly toxic heavy metals such as cadmium and lead. Avoid ingestion, inhalation and skin contact. Please wear nitrile gloves and glasses at all times and maintain extreme care during handling these chemicals. Chemical spills should be reported to the TA right away. In case of direct contact with the QDs, Please wash the affected part with cold water for at least 5 minutes. Also, you should notify the TA immediately.

The reactions for QD synthesis are conducted under inert gas and high temperatures. There is potential burn hazard for using a stirring hotplate. Caution should be taken to avoid touching the surface of a hotplate and do NOT touch the 3-neck round bottom flask during the synthesis with your fingers.

Cleaning and waste disposal:

Clean up the balance area immediately if there is any chemical spill during the measurement.

After synthesis (Part A), use a magnetic stir bar retriever to take the stir bar out of the 3-neck flask and put it into the beaker which is used to store stir bars. Then, clean up your glassware using soap water and rinse it with DI water.

Dispose all liquid waste in the waste bottle located in one of the fume hoods (TA will identify the fume hood in the lab). Dispose sharp needles, tips, and broken or used glass pipet into the red sharps container inside the fume hood. All the waste must be disposed of in the designated containers. Do not throw anything in the sink.

References:

1. Reiss, P.; Protière, M.; Li, L., Core/Shell Semiconductor Nanocrystals. _Small_ **2009**, *5 (2)*, 154-168.
2. Chen, D.; Zhao, F.; Qi, H.; Rutherford, M.; Peng, X., Bright and Stable Purple/Blue Emitting CdS/ZnS Core/Shell Nanocrystals Grown by Thermal Cycling Using a Single-Source Precursor. _Chemistry of Materials_ **2010,** *22* (4), 1437-1444.
3. Exciton Energy Shifts and Tunable Dopant Emission in Manganese-Doped Two-Dimensional CdS/ZnS Core/Shell Nanoplatelets. Davis, A. H.; Hofman, E.; Chen, K.; Li, Z.-J.; Khammang, A.; Zamani, H.; Franck, J. M.; Maye, M. M.; Meulenberg, R. W.; Zheng, W., _Chemistry of Materials_ **2019**, *31* (7), 2516-2523.
4. https://en.wikipedia.org/wiki/Core%E2%80%93shell_semiconductor_nanocrystal

Prelab Questions (10 points):

1. Discussion a) the difference of surface atoms and atoms inside the core of a crystals and b) the role of surface atoms in the nanocrystals. (4 points)
2. What type (type I, type II, or reverse type I) of core/shell structure are we forming within this lab? Explain. (3 points)
3. Discuss at least three benefits of shelling CdS QDs with a ZnS shell? (3 points)
