# Supplementary material for: An Inorganic Chemistry Laboratory Technique Course using Scaffolded, Inquiry-Based Laboratories and Project-Based Learning
Source: J Chem Educ. 2023 Aug 15;100(9):3500–8. doi: 10.1021/acs.jchemed.3c00547 (PMC10501116; doi:10.1021/acs.jchemed.3c00547)
Supplement: Supplementary file 3 — ed3c00547_si_003.pdf [file ed3c00547_si_003.pdf]

Supporting Information for

An Inorganic Chemistry Laboratory Technique Course using Scaffolded, Inquiry-Based Labs and Project-Based Learning

Chun Chu,[a] Jessica L Dewey,[b] Weiwei Zheng *[a]

[a] Department of Chemistry, Syracuse University, Syracuse, New York 13244, United States

[b] Duke Learning Innovation, Duke University, Durham, North Carolina 27708, United States

Corresponding Author

*E-mail: wzhen104@syr.edu

Lecture 6: Core/shell nanomaterials

- Why core/shell NMs
- Classification of Core/shell systems
- Synthesis of core/shell NMs

Core/Shell Semiconductor Nanocrystals.
Reiss, P.; Protière, M.; Li, L. *Small* **2009**, *5*, 154.

Core/shell nanocrystals

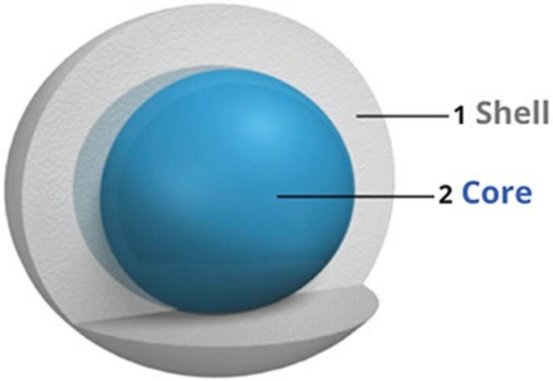

http://www.pcimag.com/articles/98223-core-shell-nanoparticle-metal-oxide-infusion

- Colloidal core/shell nanocrystals contain at least two materials in an onion-like structure
- Typical notation: core/shell, core–shell.
- Core–shell nanocrystal architecture was initially investigated in the 1990s. In 1996 Hines and Guyot-Sionnest made CdSe/ZnS core/shell nanocrystals:
- Optical properties of QDs can be substantially improved by growing an inorganic shell of a wide band gap semiconductor around the nanocrystal

Quantum yield (QY)

➢ Quantum yield (QY) is the emission efficiency of a given fluorophore.

$$QY = \frac{\#\,photons\_emitted}{\#\,photons\_absorbed}$$

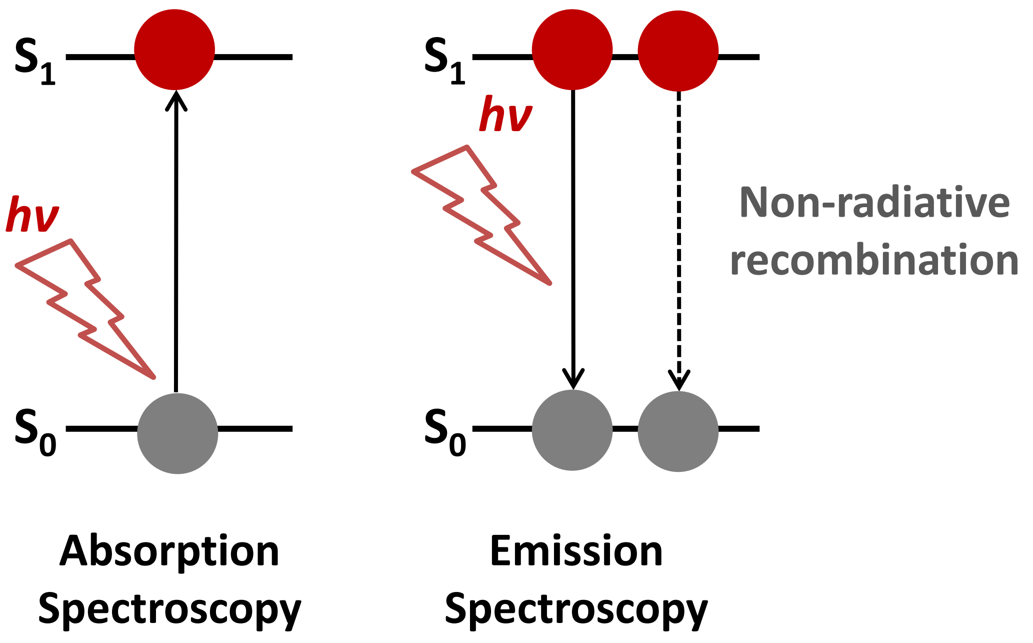

Surface defects and quantum yield

- Dangling bonds: the incomplete bonding results in atomic orbitals that point away from the surface. If the energy of the dangling orbital is within the semiconductor bandgap, electrons and holes can be trapped at the crystal surface.
- Charge carrier trapping on QDs increases the probability of non-radiative recombination, which reduces the fluorescence quantum yield.
- Surface-bound organic ligands are typically used to coordinate to surface atoms (metals) having reduced coordination number in order to passivate the surface traps.

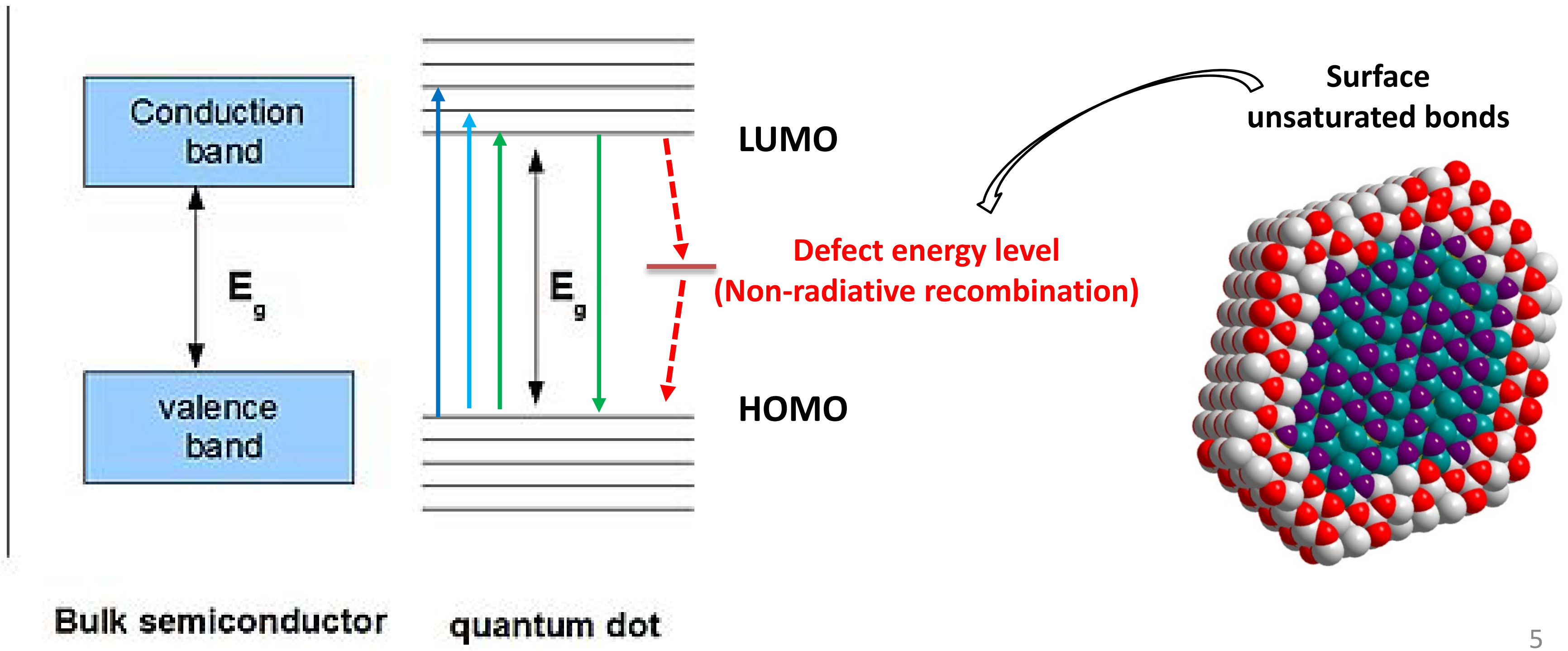

Why inorganic coating?

- Organically passivated quantum dots have low fluorescence QY due to the *incomplete passivation* of surface trap states

- Increases quantum yield (QY) by passivating both the surface anionic and cationic traps (CdSe/ZnS).

- Provides protection against environmental changes, photo-oxidative degradation.

- Provides another route for *modularity*, which enable the emission wavelength to be tuned over a wider range of wavelengths than with either individual semiconductor.

Electronic energy levels of semiconductors

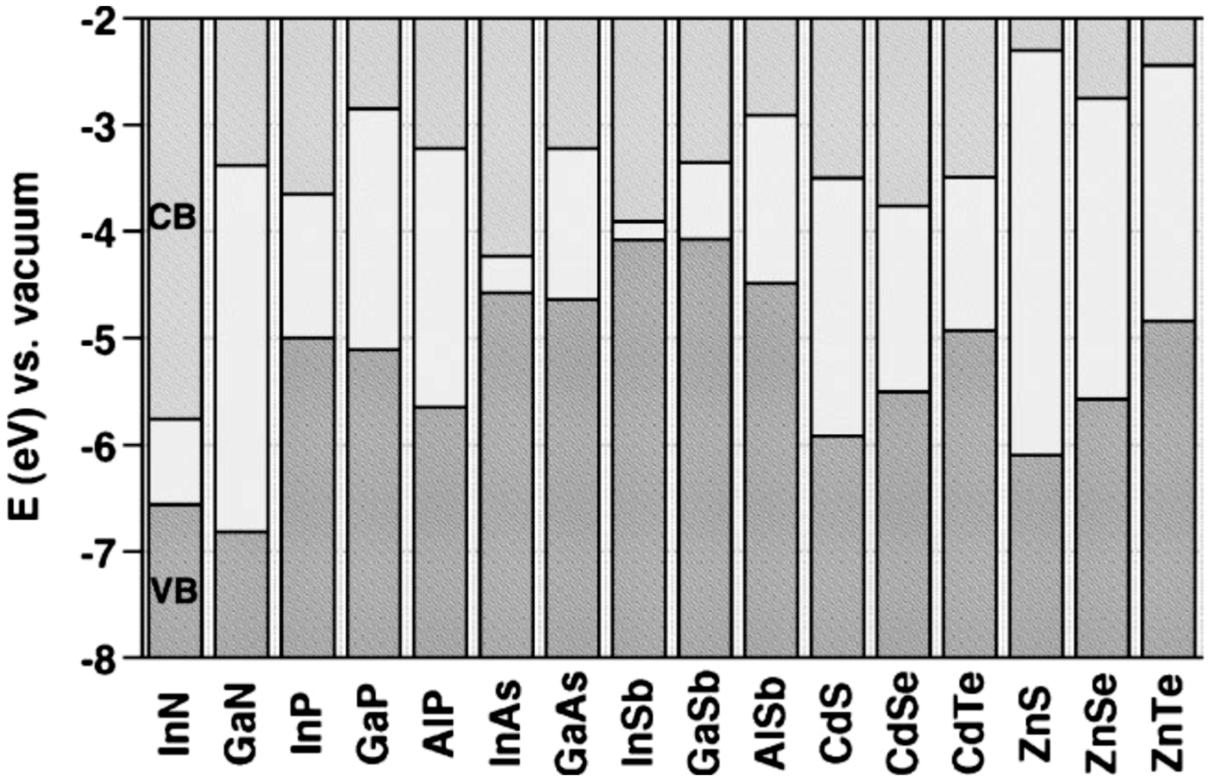

Reiss, P.; Protière, M.; Li, L. *Small* **2009**, *5*, 154.

Classification of core/shell systems

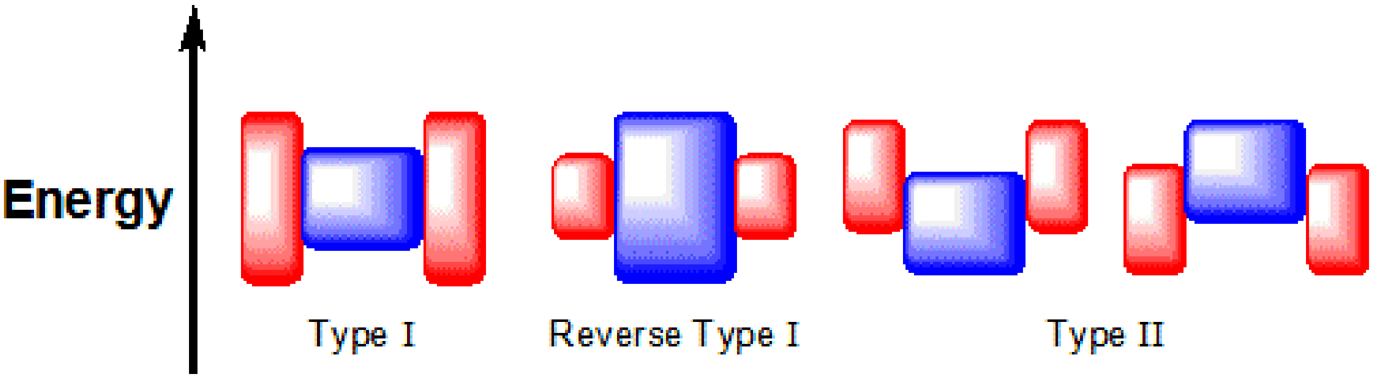

- Schematic representation of the energy-level alignment in different core/shell semiconductor NCs.
- The upper and lower edges of the rectangles correspond to the positions of the conduction- and valence-band edge of the core (center) and shell materials, respectively.

https://en.wikipedia.org/wiki/Core%E2%80%93shell_semiconductor_nanocrystal

Type I core/shell systems

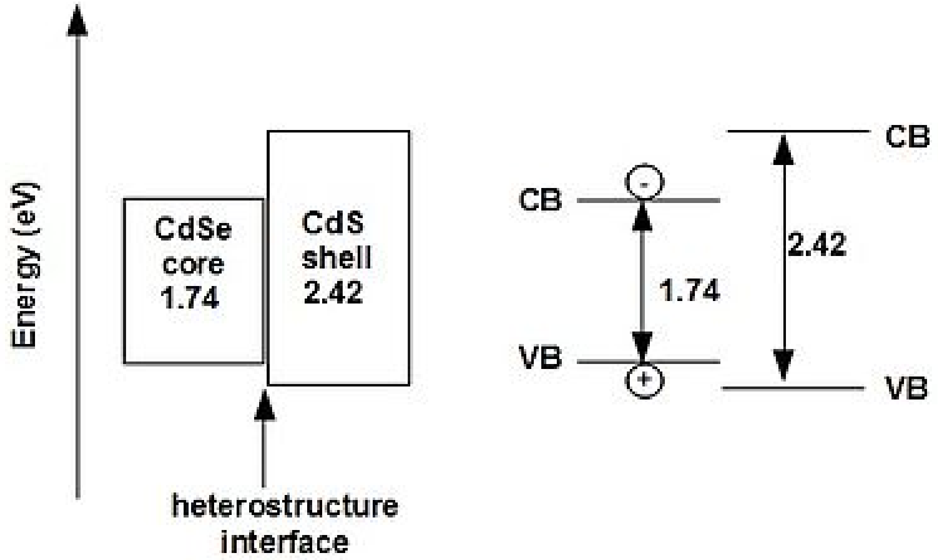

- The bandgap of the core is smaller than that of the shell. Both the conduction and valence band edges of the core lie within the bandgap of the shell, which confines both electrons and holes in the core.
- The emission wavelength due to radiative electron-hole recombination within the core is mainly unchanged or slightly redshifted compared to uncoated core NCs.
- QY increases significantly (reduces the # surface dangling bonds).
- Enhanced stability against photodegradation.
- Examples: CdSe/ZnS (1996), CdSe/CdS, and InAs/CdSe.

https://en.wikipedia.org/wiki/Core%E2%80%93shell_semiconductor_nanocrystal

Series of highly fluorescent CdSe/ZnS core/shell nanocrystals

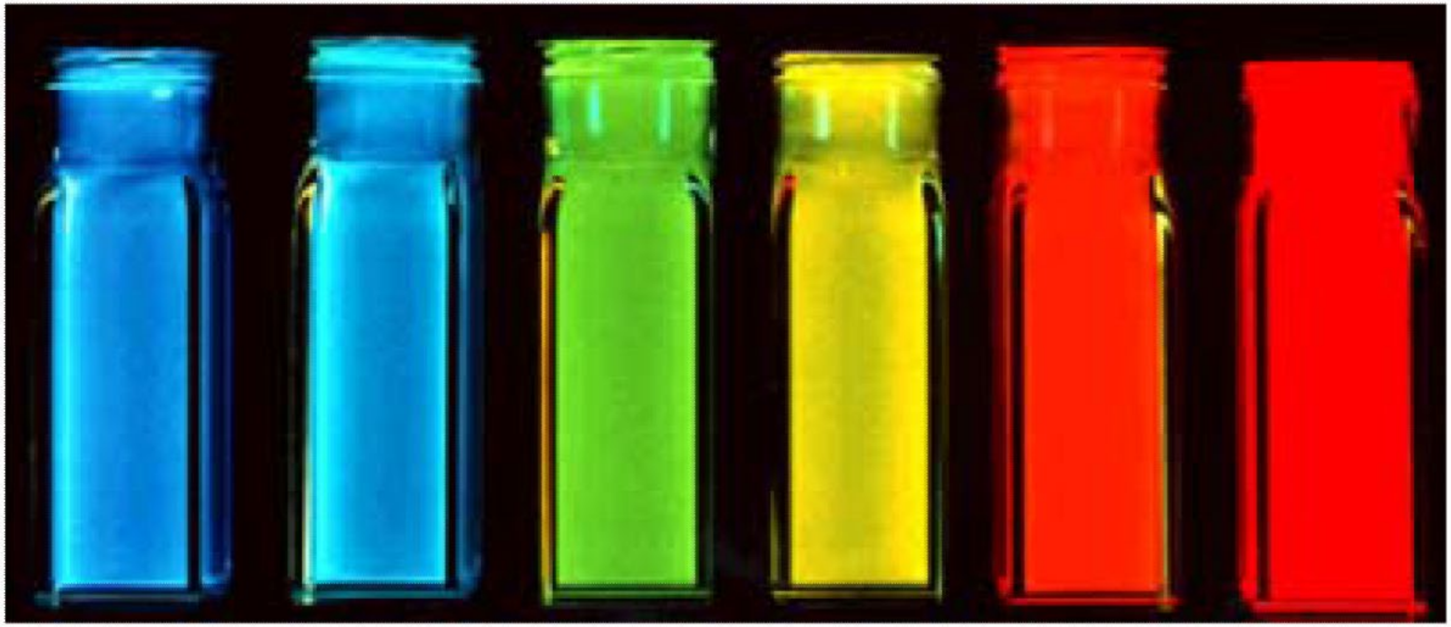

The emission can be tuned from blue through red depending upon the size of the core CdSe nanocrystal.

Rosenthal, S. J.; McBride, J.; Pennycook, S. J.; Feldman, L. C. *Surf. Sci. Rep.* **2007**, *62*, 111.

Optical properties of C/S QDs

Organic dyes

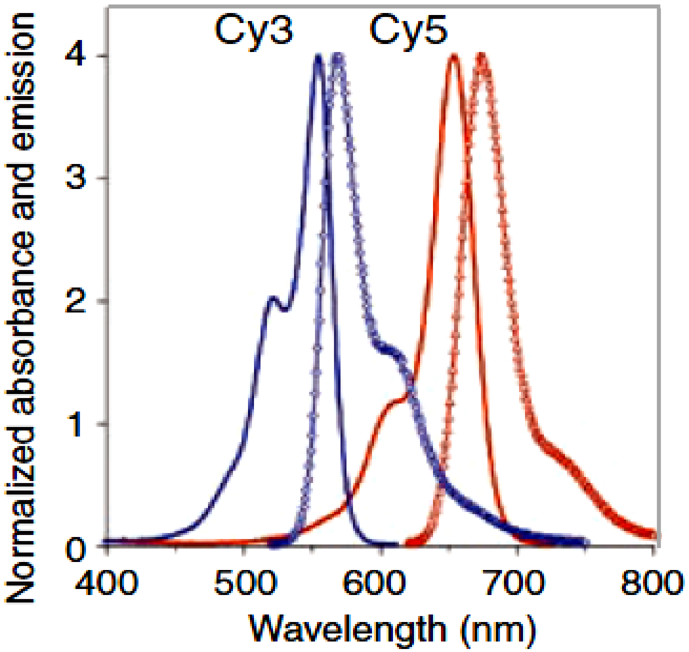

Semiconductor NCs (QDs)

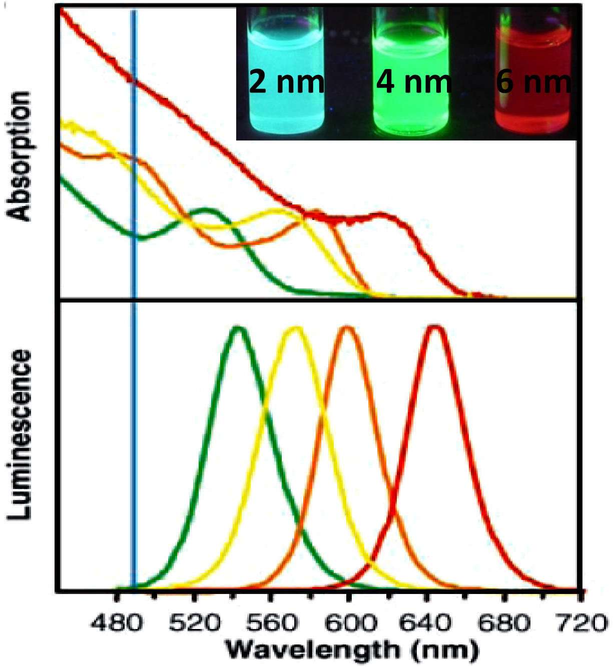

Features of C/S QDs :

- ➢ High emission quantum yields
- ➢ Broad absorption spectra
- ➢ Tunable narrow emission spectra
- ➢ Excellent photostability

Resch-Genger, U. *et. al. Nat. Methods* **2008,** *5,* 763.

Weiss, S. *et. al. Science* **2005,** *307*, 538.

Reverse type I core/shell systems

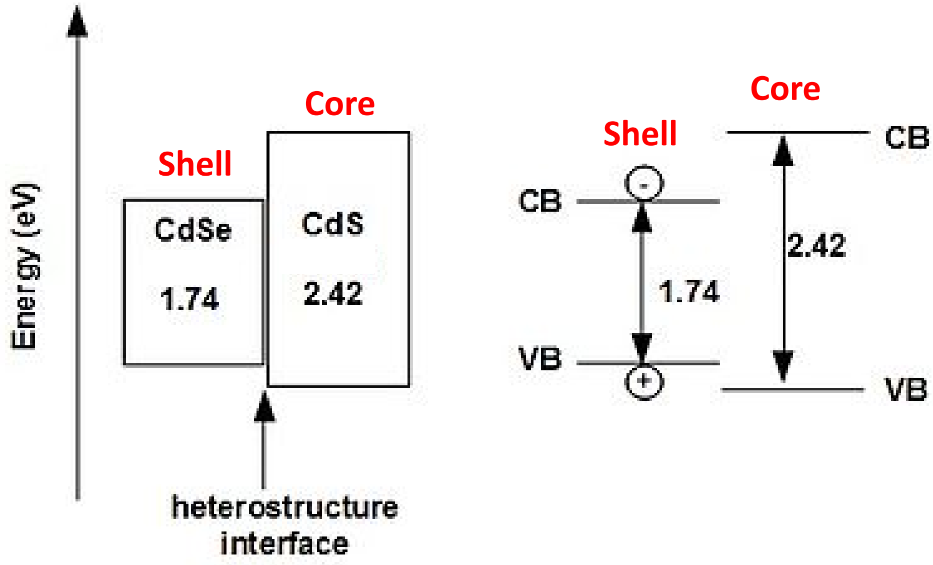

- The core has a wider bandgap than the shell, and the conduction and valence band edges of the shell lie within those of the core.
- The lowest available exciton energy separation occurs when the charge carriers are localized in the shell. Generally, a significant red-shift of the bandgap with the shell thickness is observed.
- Examples: CdS/HgS, CdS/CdSe, and ZnSe/CdSe.

https://en.wikipedia.org/wiki/Core%E2%80%93shell_semiconductor_nanocrystal

Type II core/shell systems

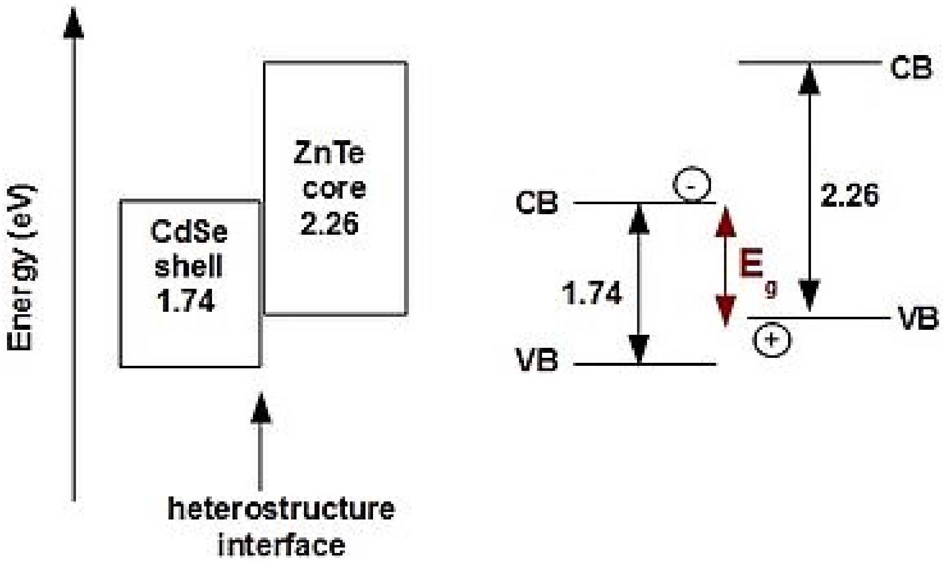

- The valence and conduction band edge of the core are both lower or higher than the band edges of the shell.
- The emission wavelength will be determined by the energy difference between these occupied states, which will be at a lower energy than either of the individual bandgaps. The emission wavelength can be significantly red shifted compared to the unpassivated core.
- Promising candidates for photovoltaic applications due to the separate of the electrons and holes and fast electron transport.
- Examples: ZnTe/CdSe, CdTe/CdSe, CdS/ZnSe.

https://en.wikipedia.org/wiki/Core%E2%80%93shell_semiconductor_nanocrystal

General considerations for the design of core/shell systems

- Shell materials should crystallize in the same structure and exhibit a small **lattice mismatch**
- Precursors for shell growth should have appropriate reactivity and selectivity (no side reactions)

Table 1. Material parameters of selected bulk semiconductors.[20,21]

Material	Structure [300K]	Type	E_{gap} [eV]	Lattice parameter [Å]	Density [kg m^{-3}]
ZnS	Zinc blende	II–VI	3.61	5.41	4090
ZnSe	Zinc blende	II–VI	2.69	5.668	5266
ZnTe	Zinc blende	II–VI	2.39	6.104	5636
CdS	Wurtzite	II–VI	2.49	4.136/6.714	4820
CdSe	Wurtzite	II–VI	1.74	4.3/7.01	5810
CdTe	Zinc blende	II–VI	1.43	6.482	5870
GaN	Wurtzite	III–V	3.44	3.188/5.185	6095
GaP	Zinc-blende	III–V	2.27	5.45	4138
GaAs	Zinc blende	III–V	1.42	5.653	5318
GaSb	Zinc blende	III–V	0.75	6.096	5614
InN	Wurtzite	III–V	0.8	3.545/5.703	6810
InP	Zinc blende	III–V	1.35	5.869	4787
InAs	Zinc blende	III–V	0.35	6.058	5667
InSb	Zinc blende	III–V	0.23	6.479	5774
PbS	Rocksalt	IV–VI	0.41	5.936	7597
PbSe	Rocksalt	IV–VI	0.28	6.117	8260
PbTe	Rocksalt	IV–VI	0.31	6.462	8219

4% lattice mismatch

3.2% lattice mismatch

Reiss, P.; Protière, M.; Li, L. *Small* **2009**, *5*, 154.

One pot method: CdSe/ZnS NCs

- Synthesis of core nanocrystals
- Add shell stock solution in steps at lower temperature

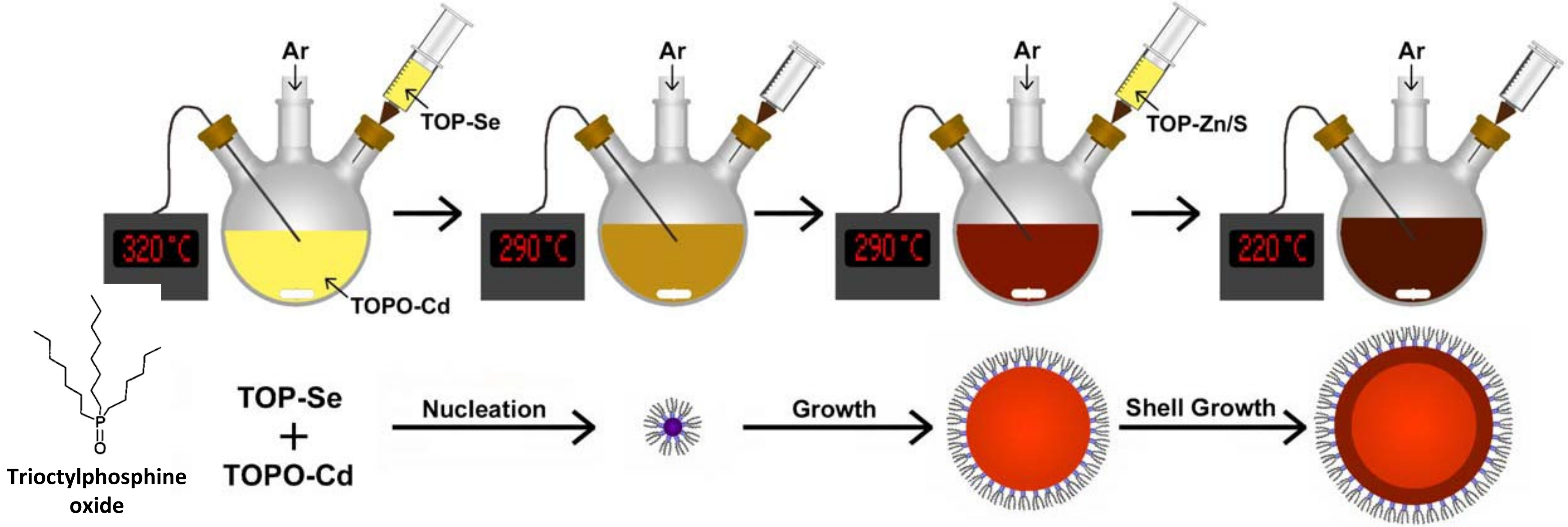

- The growth condition control
  - no homogeneous nucleation occur and only grow on the surface
    - temperature control
  - concentration control: not high enough for nucleation but high enough for growth
    - drop wise addition or in steps addition

Bailey, R. E.; Smith, A. M.; Nie, S. *Physica E: Low-dimensional Systems and Nanostructures* **2004,** *25,* 1.

Two pot synthesis of core/shell nanocrystals

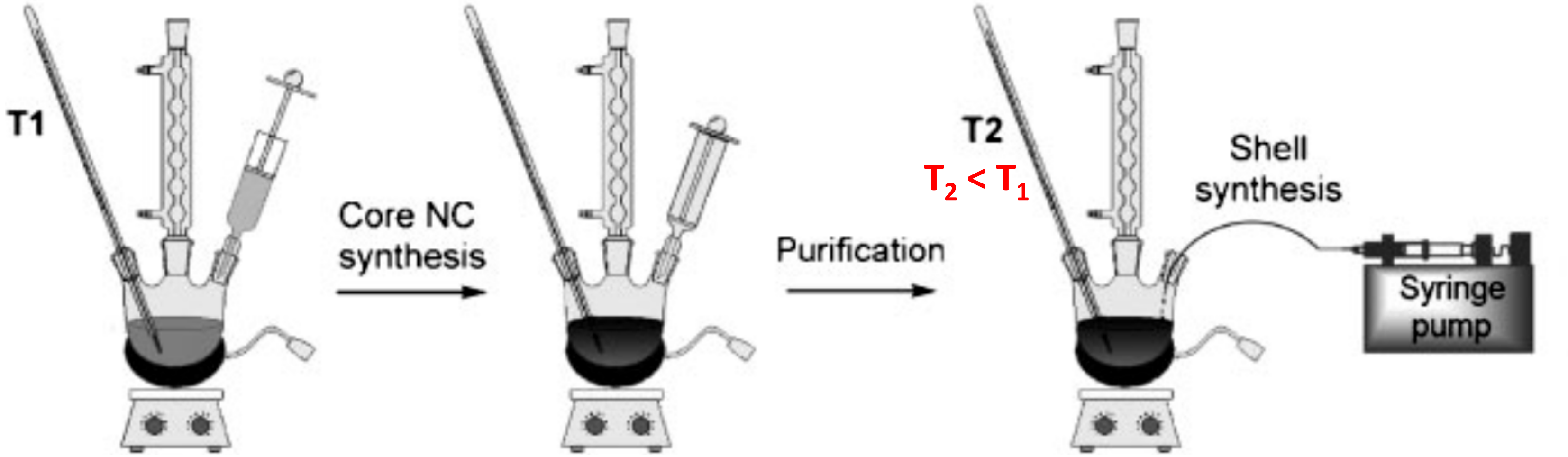

- Synthesis of core nanocrystals
- These core nanocrystals were isolated as powders and then redispersed in organic solvents
- Add shell stock solution in steps at lower temperature

Reiss, P.; Protière, M.; Li, L. *Small* **2009**, *5*, 154.

Post lab questions (10 points)

1. What is the surface effects of nanomaterials? Search online and discuss how physical properties (such as optical, electrical, mechanical, etc.) of nanomaterials change when compared to macroscopic systems. (5 points)

2. Calculate the lattice mismatch between CdS core and ZnS shell (both are in cubic (zinc blend) structure. (2 points)

3. Discuss the difference between one-pot and two-pot synthesis for core/shell nanocrystals. And explain which synthetic method is used in our experiment. (3 points)

Questions?
