# Supplementary material for: An Inorganic Chemistry Laboratory Technique Course using Scaffolded, Inquiry-Based Laboratories and Project-Based Learning
Source: J Chem Educ. 2023 Aug 15;100(9):3500–8. doi: 10.1021/acs.jchemed.3c00547 (PMC10501116; doi:10.1021/acs.jchemed.3c00547)
Supplement: Supplementary file 4 — ed3c00547_si_004.pdf [file ed3c00547_si_004.pdf]

Supporting Information for

An Inorganic Chemistry Laboratory Technique Course using Scaffolded, Inquiry-Based Labs and Project-Based Learning

Chun Chu,[a] Jessica L Dewey,[b] Weiwei Zheng *[a]

[a] Department of Chemistry, Syracuse University, Syracuse, New York 13244, United States

[b] Duke Learning Innovation, Duke University, Durham, North Carolina 27708, United States

Corresponding Author

*E-mail: wzhen104@syr.edu

The Application of Nanoparticles to Enhance Latent Fingerprints

Department of Chemistry

Syracuse University 23’

Background

- Latent print biological components
- Traditional methods are destructive
- Nanoparticles are new leading latent print research

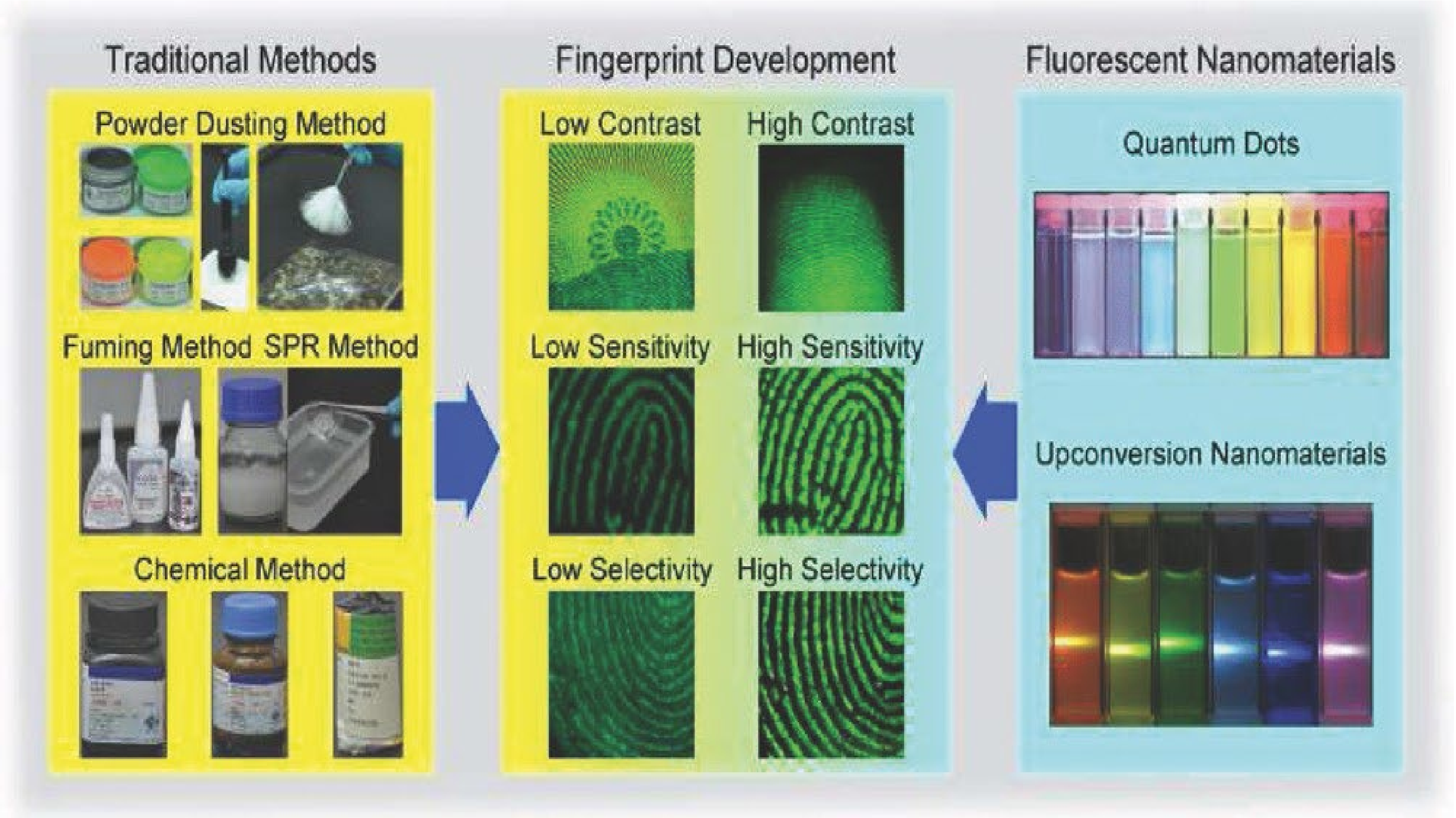

Comparison of traditional techniques to nanoparticles (1)

Properties and Significance

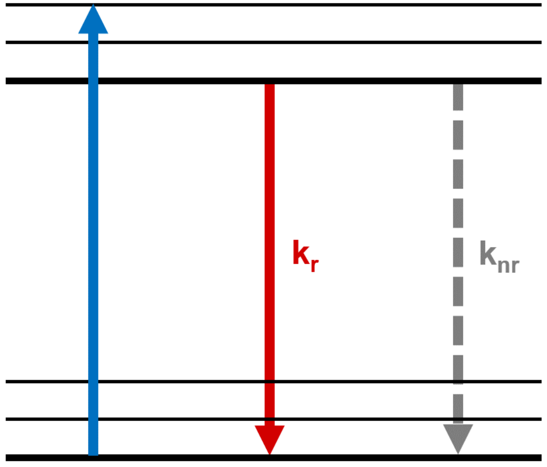

Quantum Yield (Φ)

$$\Phi = \frac{N_{Photons\ Emitted}}{N_{Photons\ Absorbed}}$$

$$= \frac{k_r}{k_r + \sum k_{nr}}$$

High quantum yield (2)

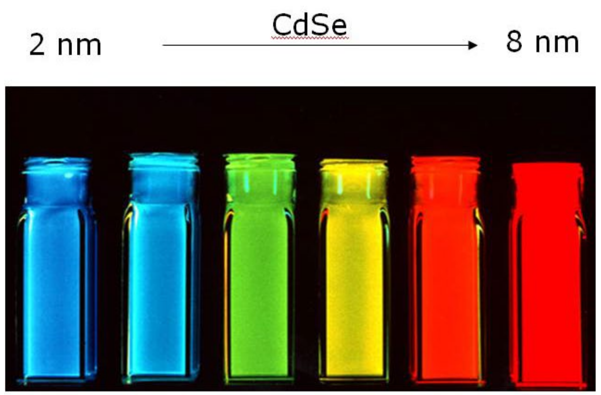

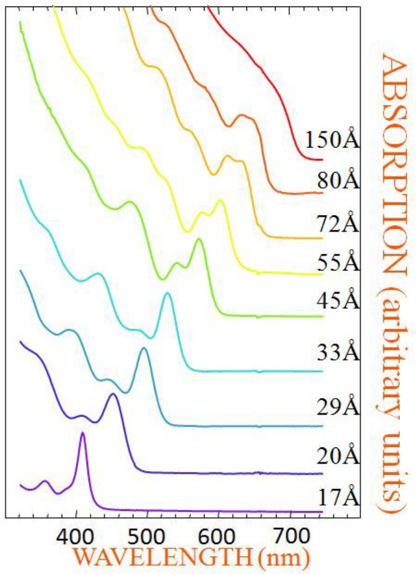

Size-dependent properties (4)

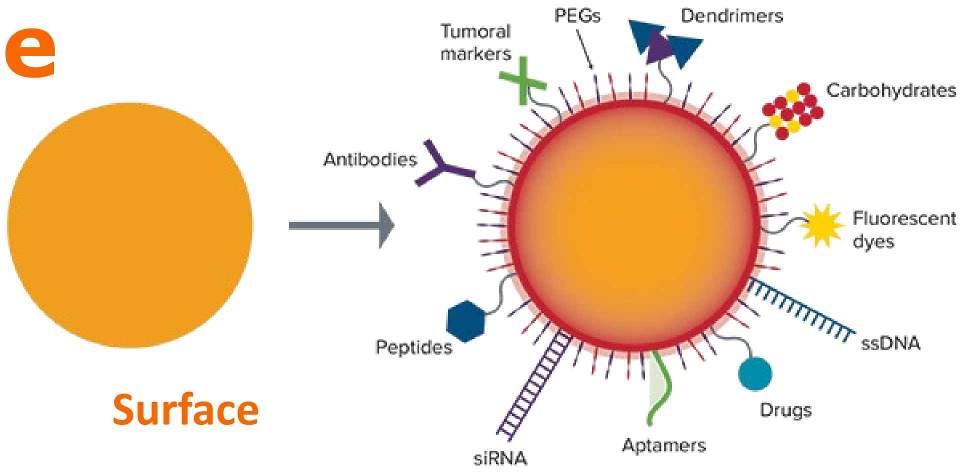

Surface Functionalization (3)

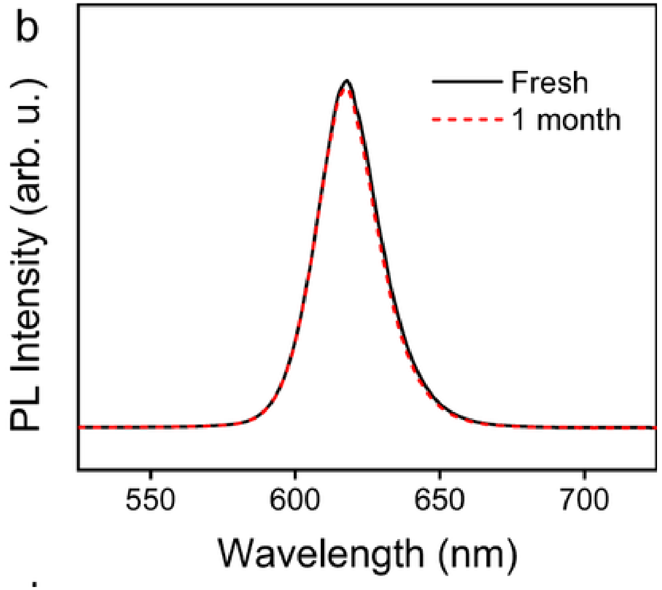

Stability and Fluorescence (5)

Current research

- Synthesized 3nm nanoparticles
- Silicon wafers immersed in 0.04% petroleum ether solution of CdSe/ZnS
- Illuminated under UV light
- Aggregation on fingerprint ridges

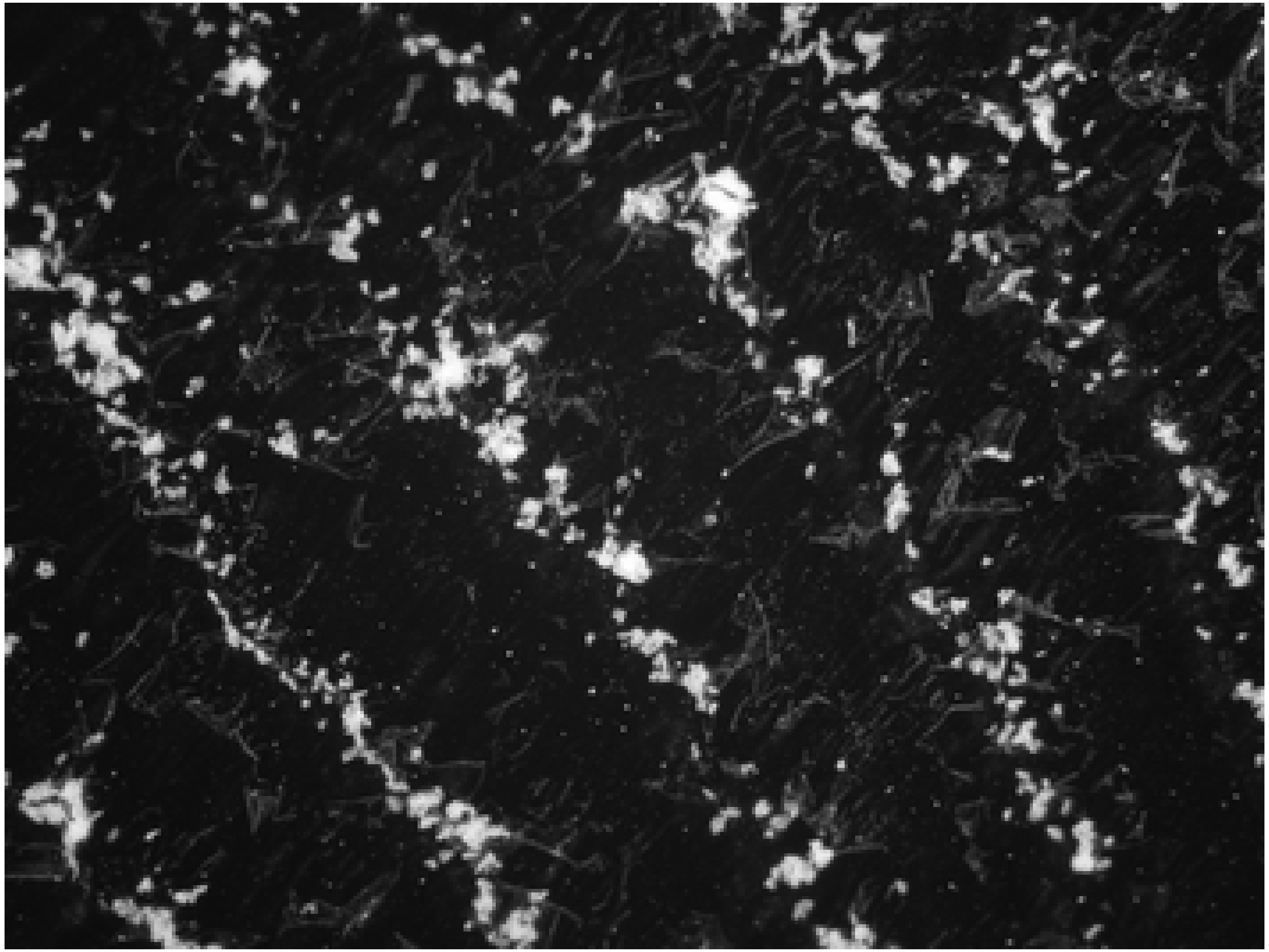

Latent fingerprint enhanced with CdSe/ZnS nanoparticles (6)

Proposed research

- ➢ Ligand exchange method
- ➢ Functionalization of the nanoparticles
- ➢ Fatty acids will bind to sebaceous secretions

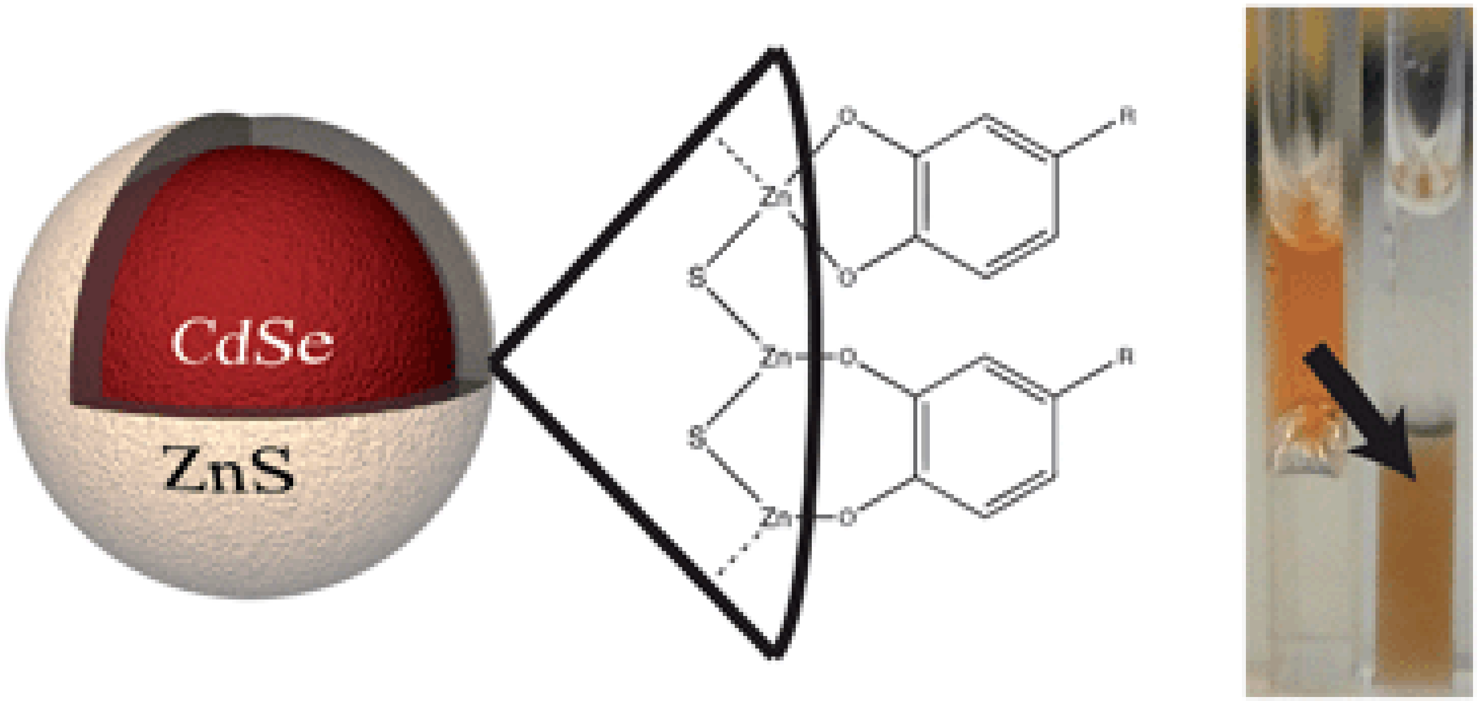

Ligand exchange with catechol molecules (7)

Synthesis of CdSe nanoparticle core (6)

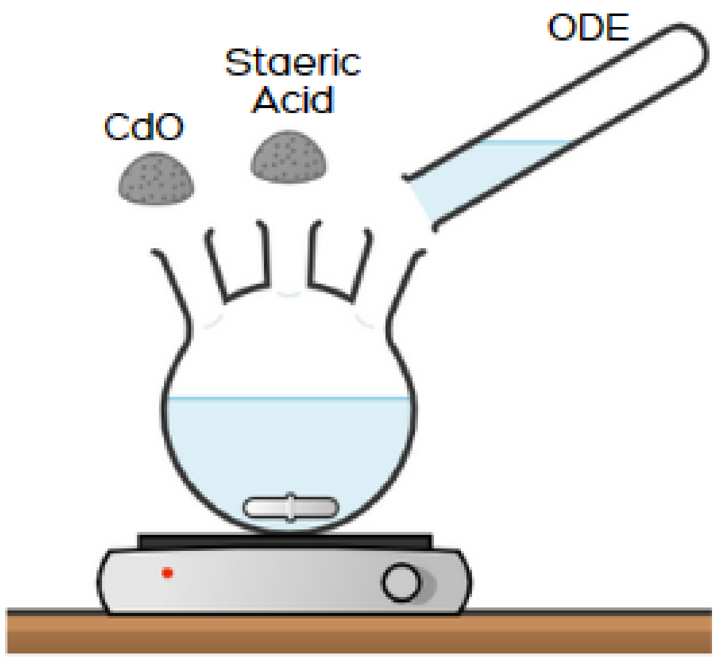

Heat the solution to

200 degrees C

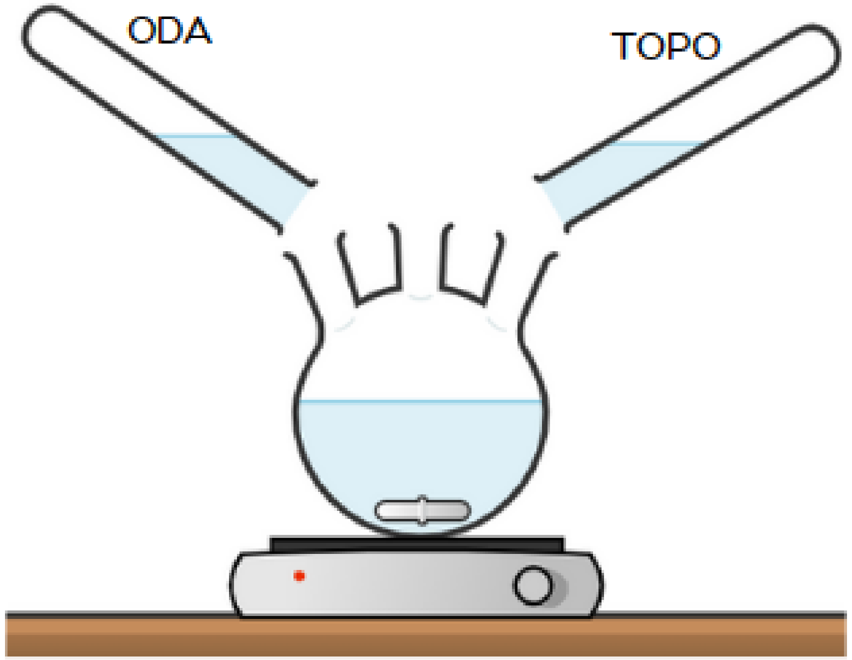

Add at room temperature,

Then reheat to 280 degrees C

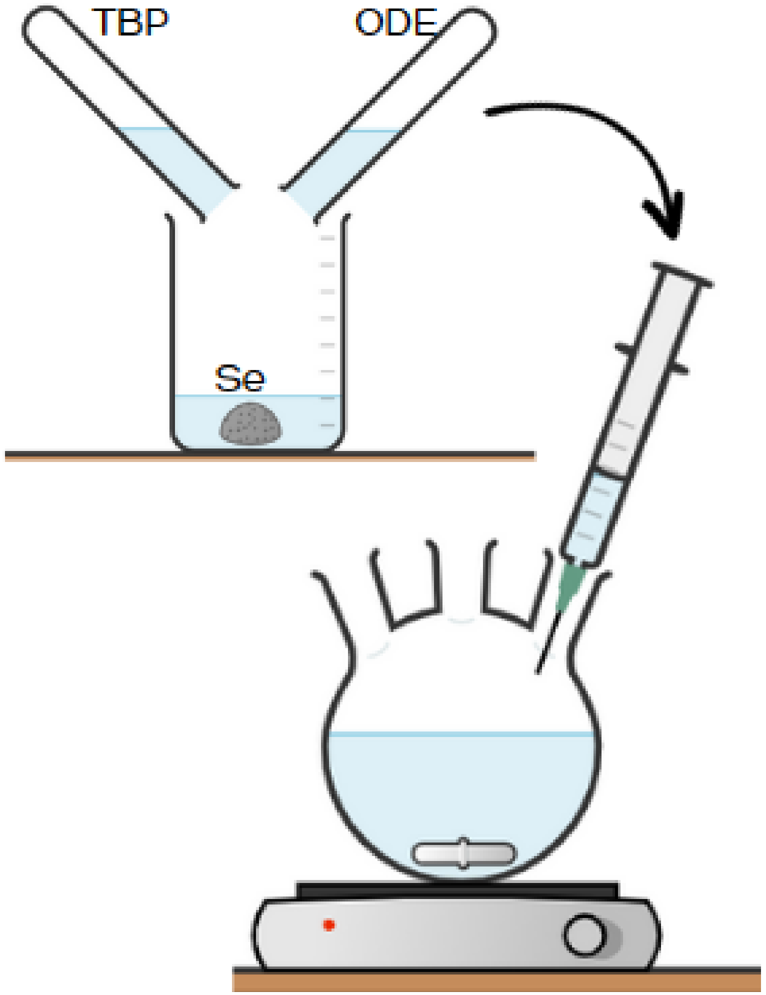

Reduce temperature to 250 degrees C

Extraction of CdSe nanoparticle core (6)

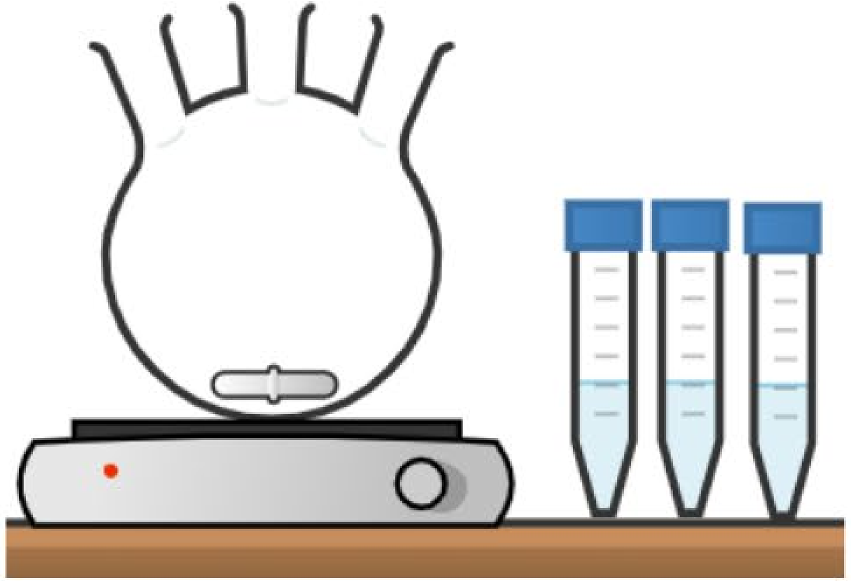

Transfer the solution into centrifuge tubes with Hexane

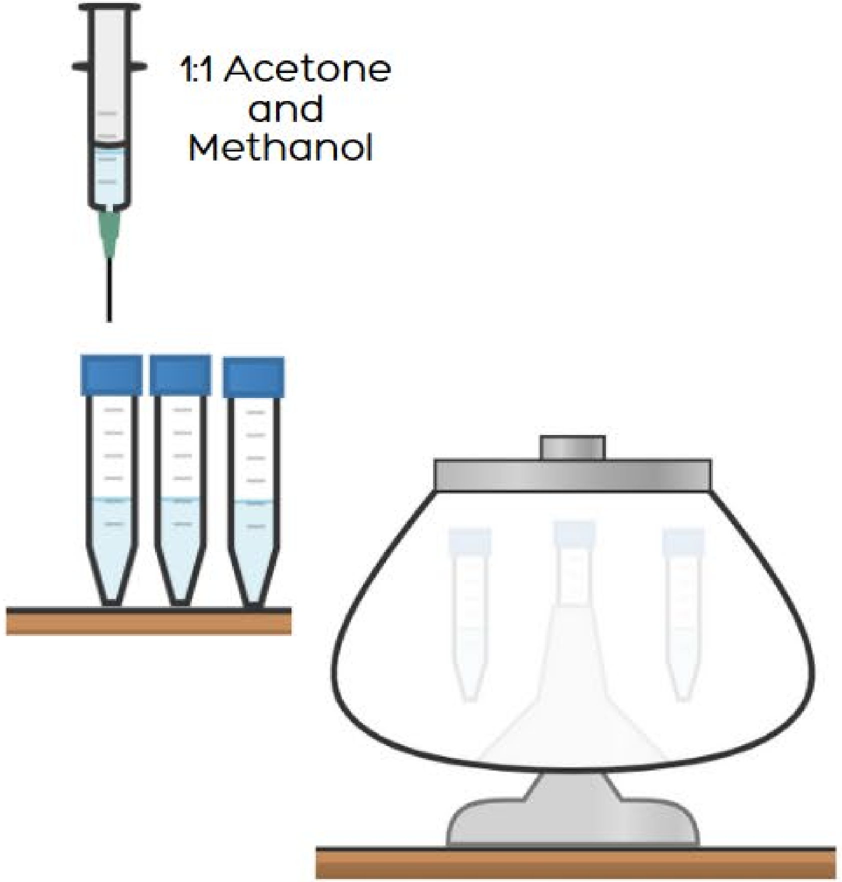

Centrifuge for 5 minutes at 500rpm

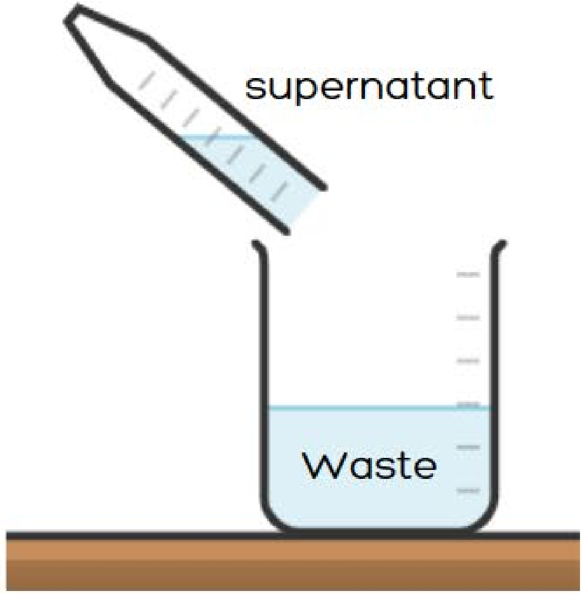

Remove supernatant, then repeat last steps

Synthesis of ZnS nanoparticle shell (6)

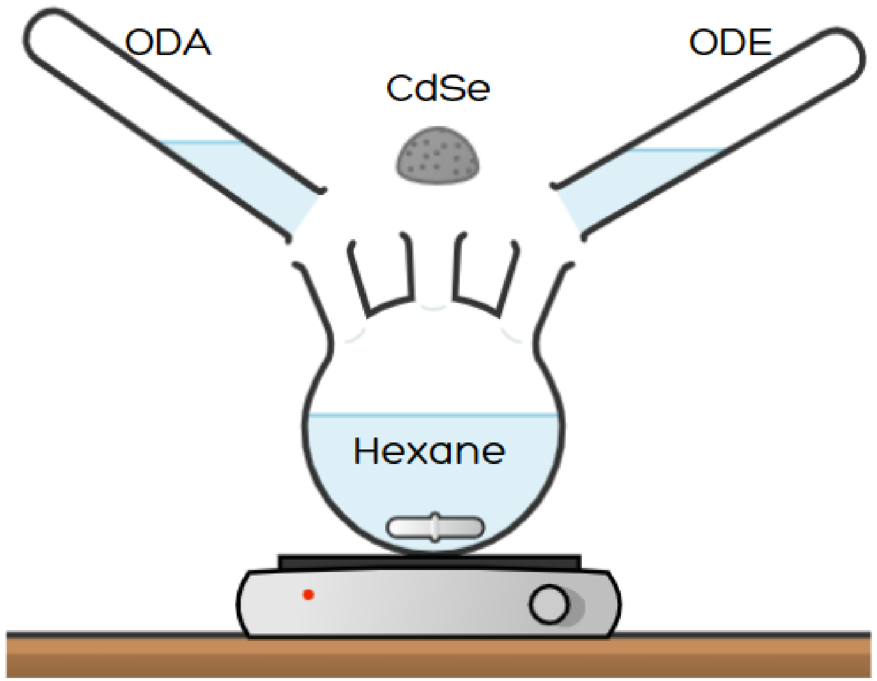

Heat the solution to

200 degrees C

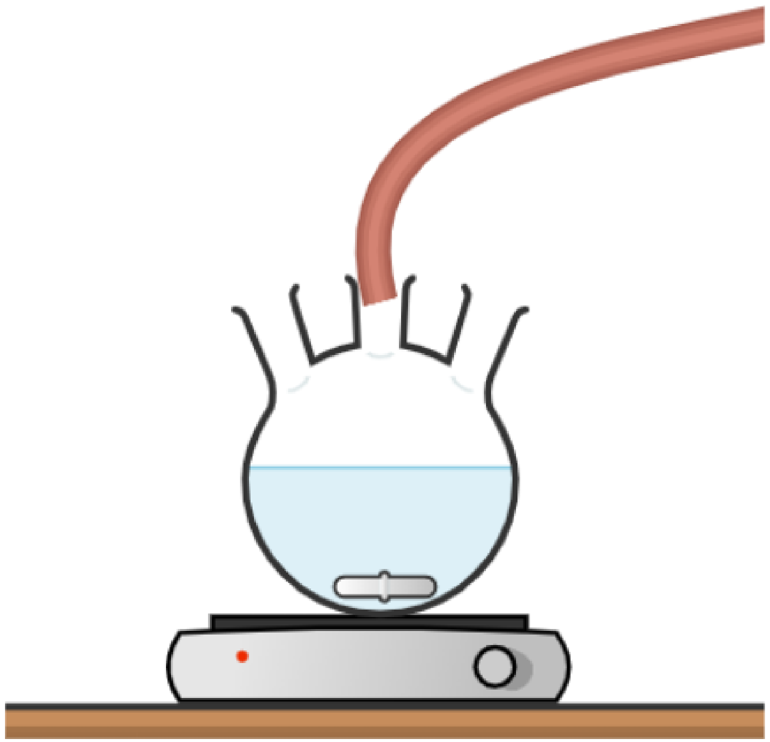

Use mechanical pump to discard hexane

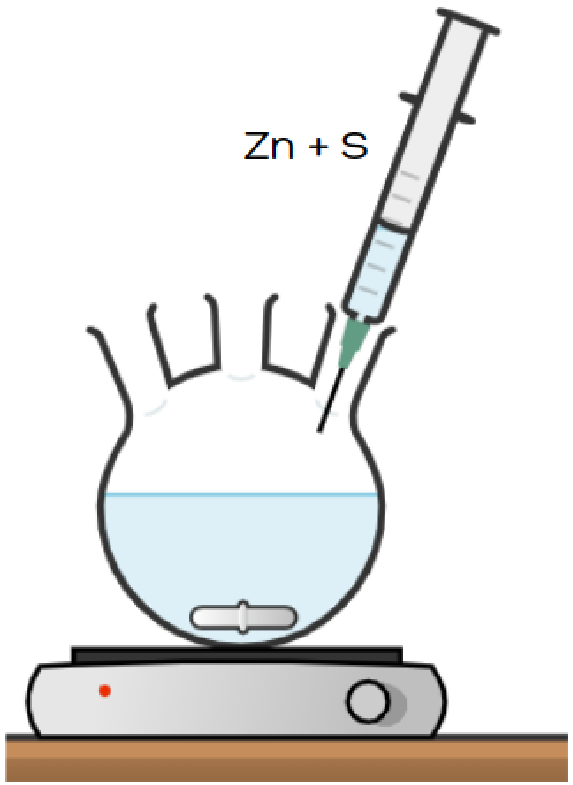

Reduce to 250 degrees C

Extraction of CdSe/ZnS nanoparticles (6)

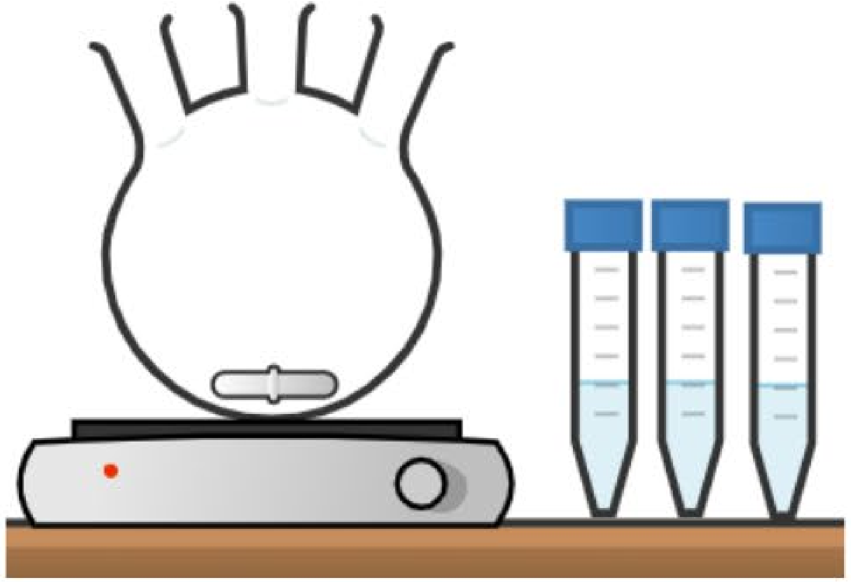

Transfer the solution into centrifuge tubes with Hexane

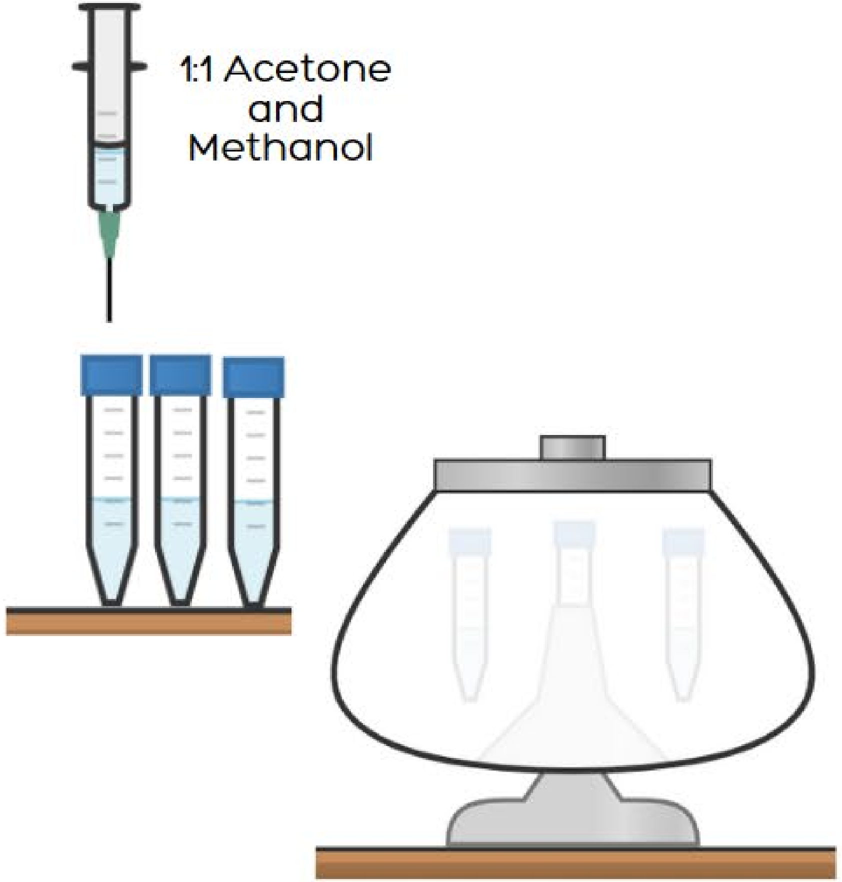

Centrifuge for 5 minutes at 500rpm

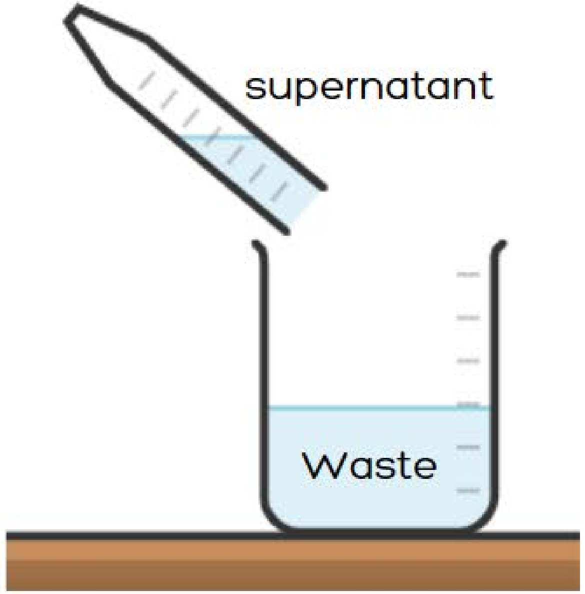

Remove supernatant, then repeat last steps

Optical properties of CdSe/ZnS nanoparticles (6)

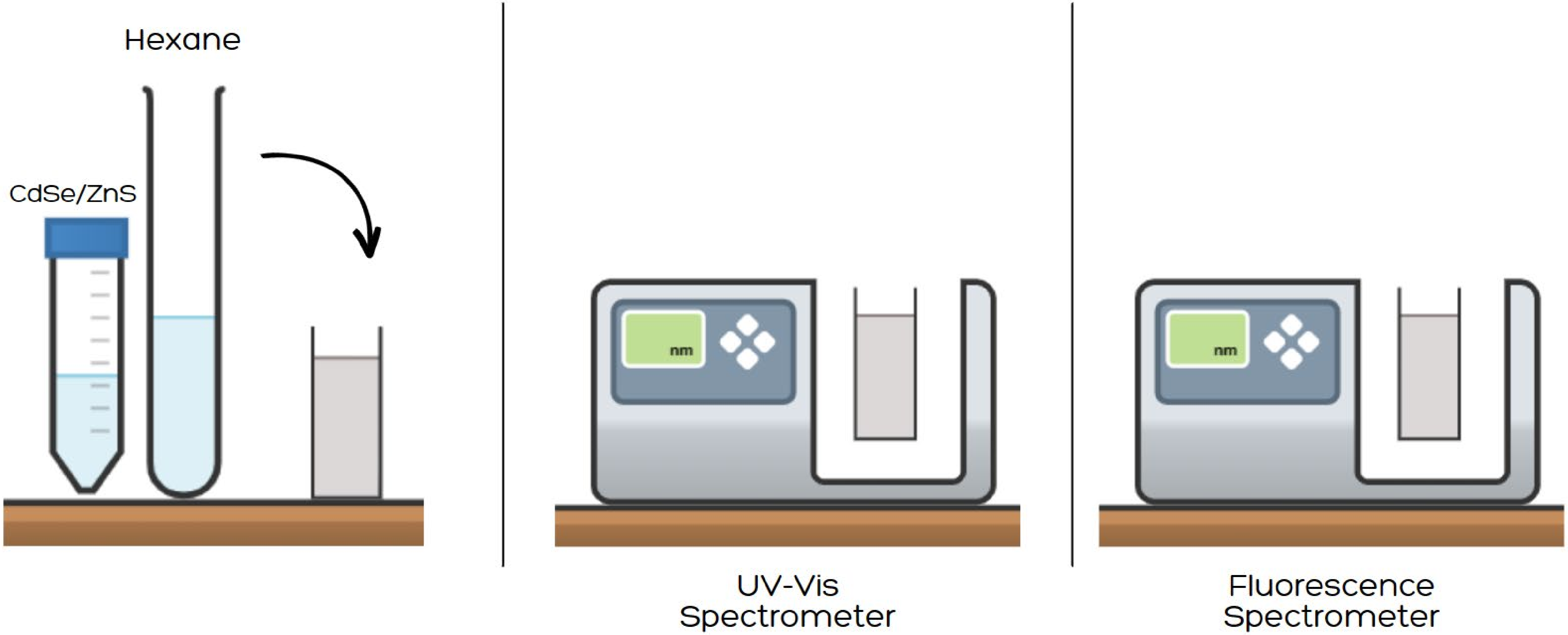

Collect the optical properties of CdSe/ZnS in hexane

Ligand Exchange *

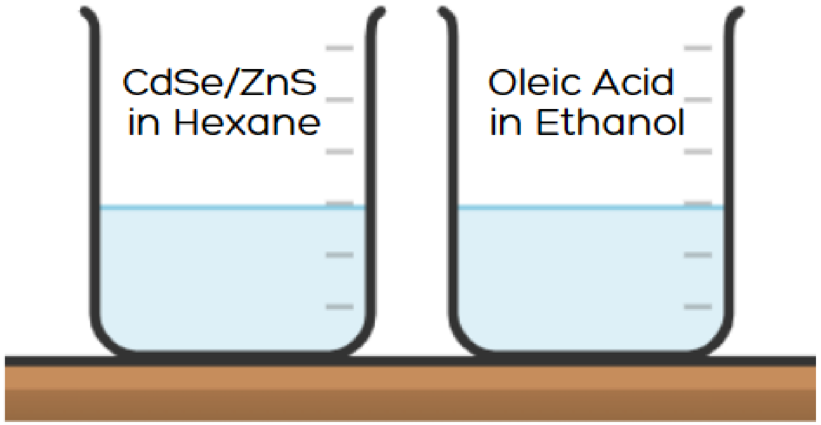

Create the stock solutions and combine 1:1

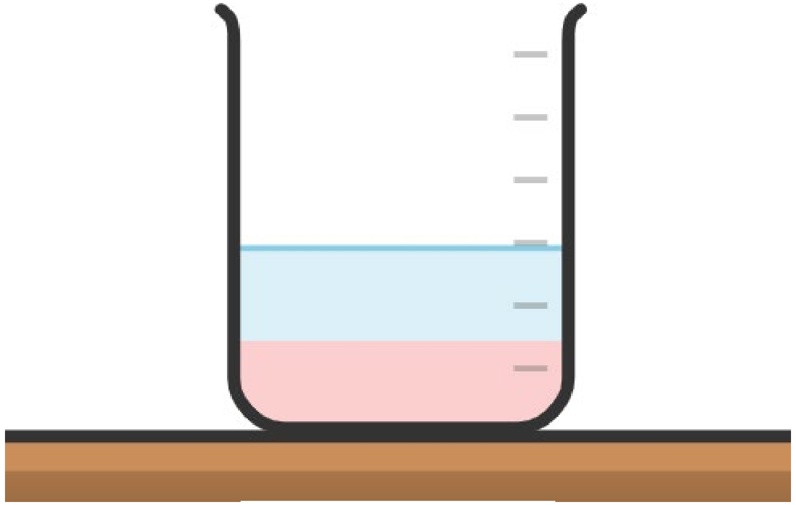

Shake the two together before letting sit for 5 minutes

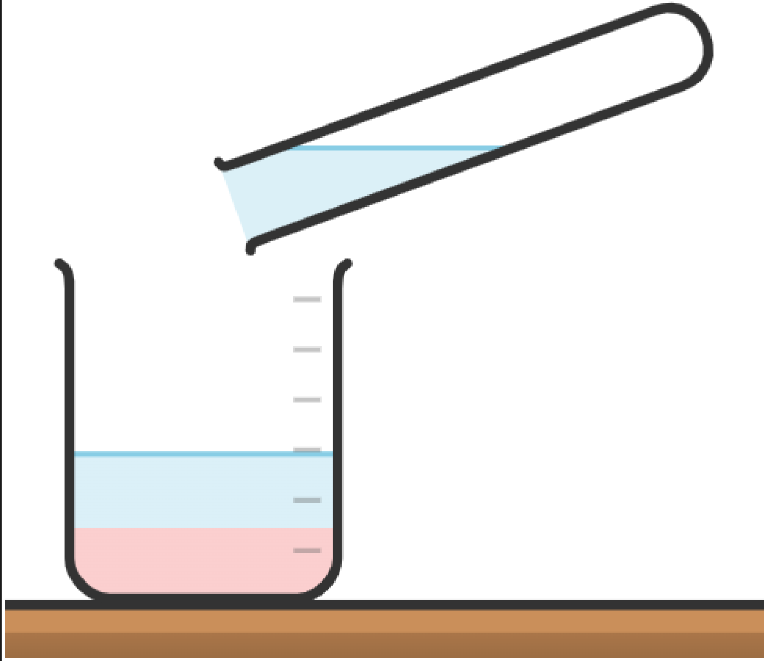

Add ethanol

Ligand Exchange *

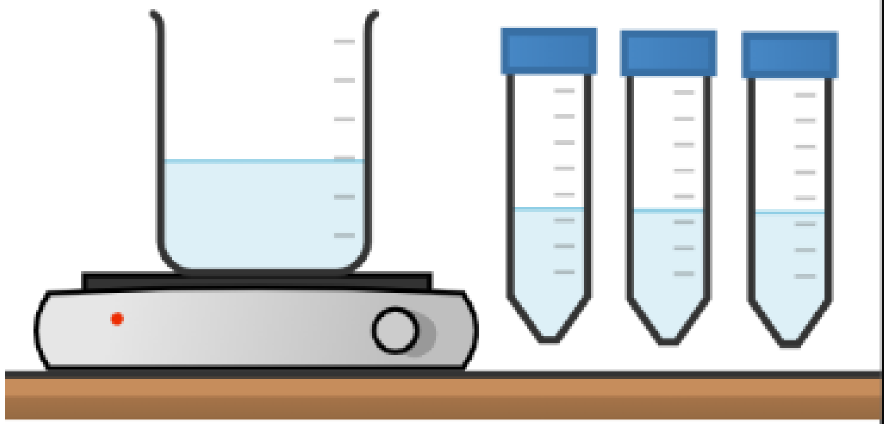

Transfer the solution into centrifuge tubes

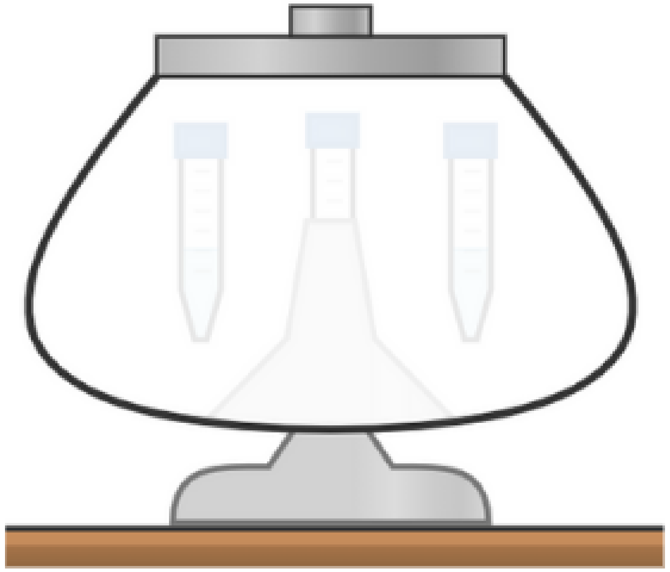

Centrifuge for 5 minutes at 500rpm

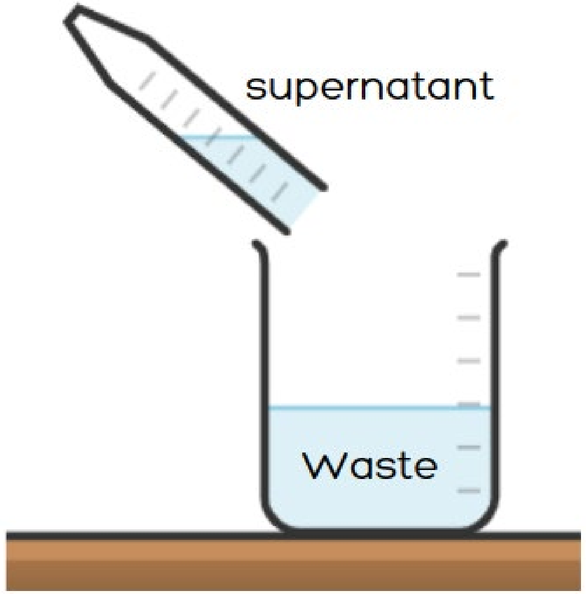

Remove the supernatant

Visualization *

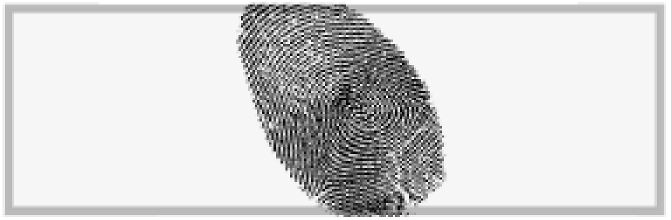

Deposit latent print on silicon wafer

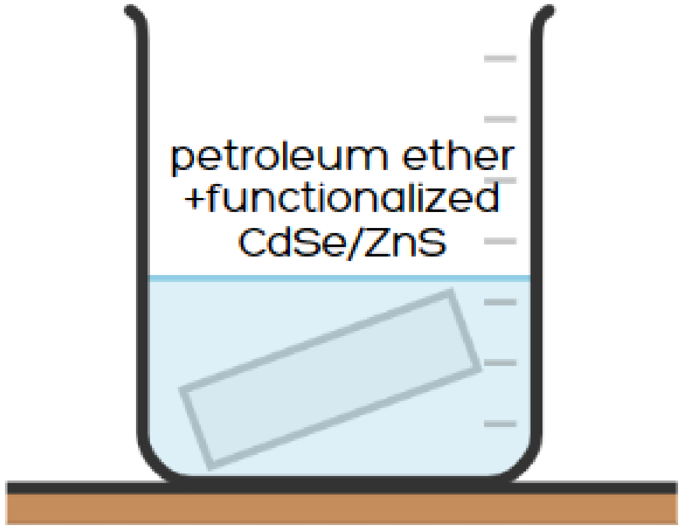

Deposit wafer into petroleum ether + functionalized CdSe/ZnS solution

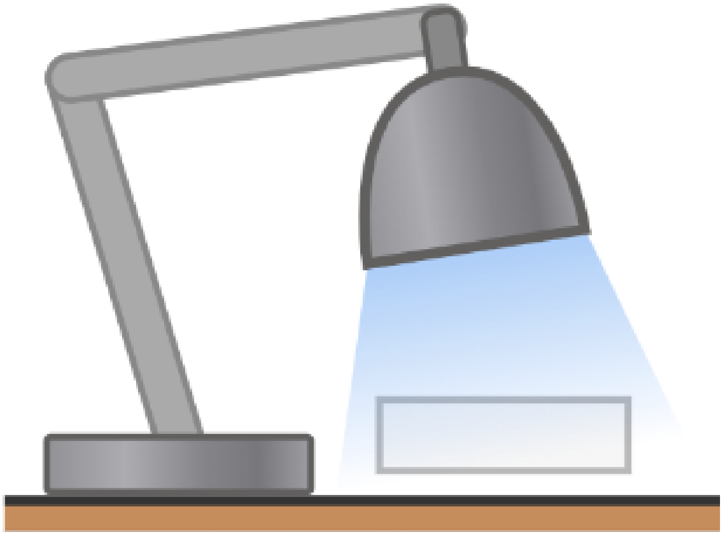

Illuminate the print with UV light

Expected Results

- Functionalized nanoparticles aggregate to sebaceous ridges
- Illumination under light source
- Stronger latent print resolution
- CdSe/ZnS nanoparticles vs functionalized CdSe/ZnS nanoparticles visualization

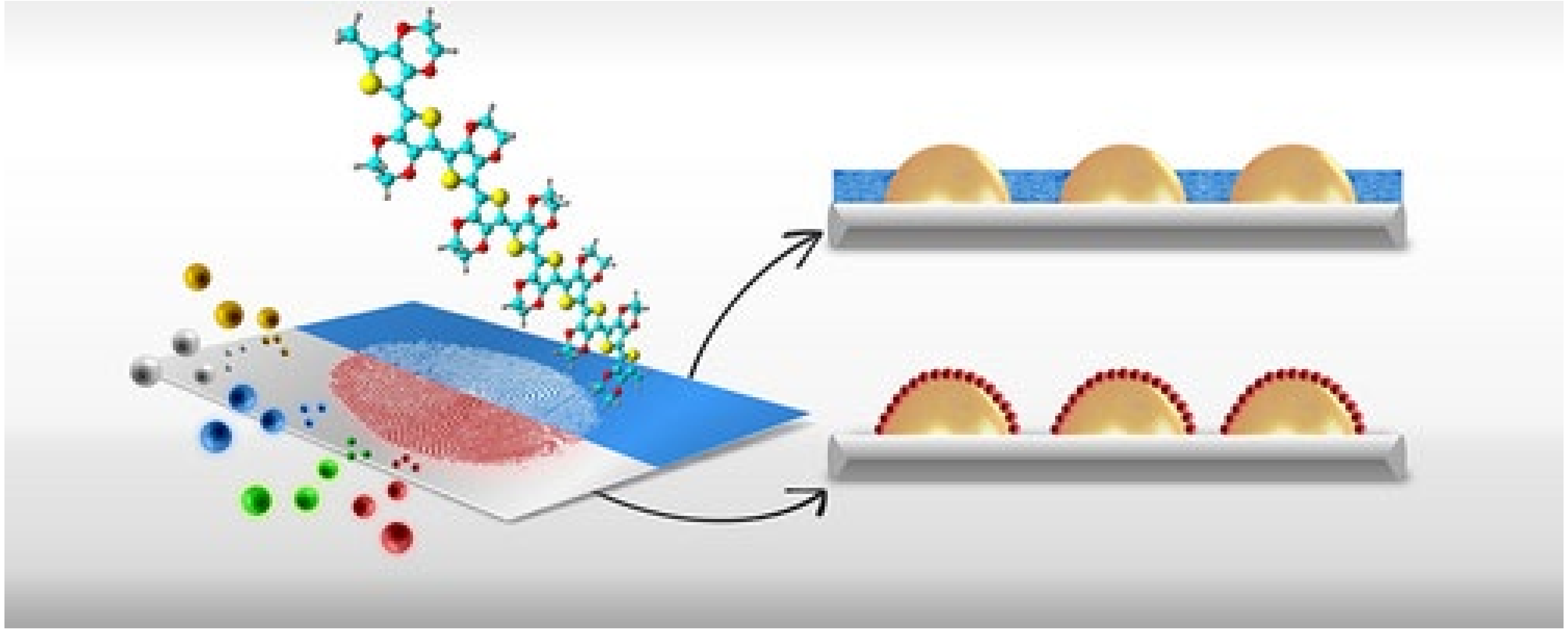

Nanoparticles adhering to sebaceous ridges (10)

Conclusion

- Reliable and stable latent print visualization
- Prevents fingerprint damage
- Future work in functionalization (Other fatty acids? Other nanoparticles?)
- Optimization of the technique

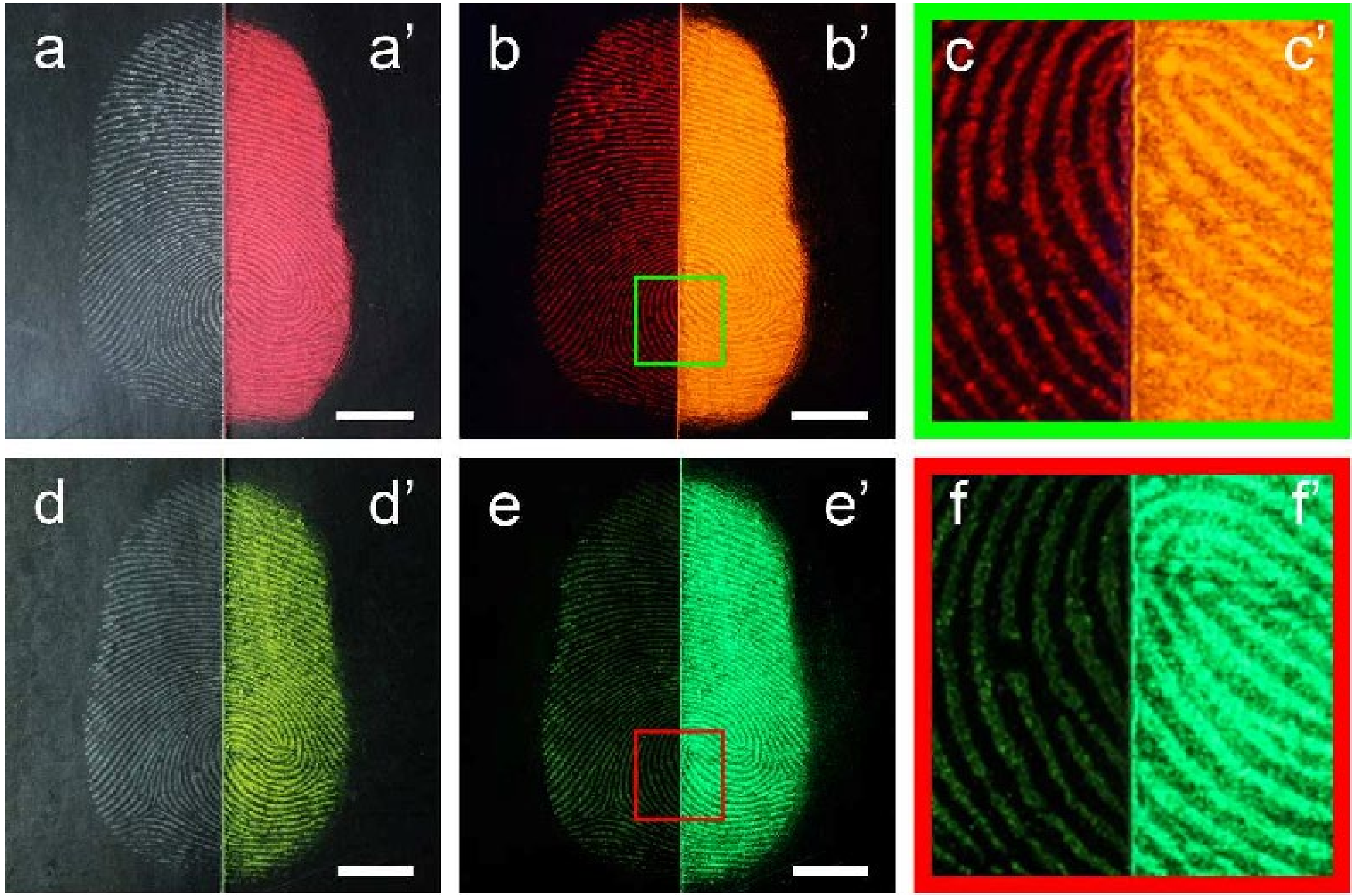

Nanoparticles adhering to sebaceous ridges (11)

References

1. Sarker, S., Sultana, S., Zaman, S., Nahar, T., Sharmin, E., Rahman, M., ... & Haque, M. M. (2014). Solvothermal synthesis of CdSe/ZnS core–shell quantum dots and their applications in DNA detection and cell imaging. RSC Advances, 4(13), 65245-65254.

2. Molecular Devices. (2018). Spectral signature analysis of surface functionalized nanoparticles. Application Note. Retrieved from https://www.moleculardevices.com/en/assets/app-note/br/spectral-signature-analysis-of-surface-functionalized-nanoparticles

3. Edinburgh Instruments. (n.d.). What is quantum yield? Retrieved from https://www.edinst.com/us/blog/what-is-quantum-yield/

4. Forensic Science Simplified. (n.d.). How are latent prints found? Retrieved from https://www.forensicsciencesimplified.org/prints/how.html

5. Chen, X., Zhou, Y., Peng, X., & Yoon, J. (2017). Fluorescent and colorimetric probes for detection of latent fingerprints. Advanced Functional Materials, 27(8), 1605597.

6. Sametband, M., Shweky, I., Banin, U., Mandler, D., & Almog, J. (2007, February 19). Application of nanoparticles for the enhancement of latent fingerprints. Chemical Communications. Retrieved March 29, 2023, from https://pubs.rsc.org/en/content/articlehtml/2007/cc/b618966k

7. Yu, W. W., & Peng, X. (2003). Formation of high-quality CdTe, CdSe, and CdS nanocrystals using CdO as precursor. Journal of the American Chemical Society, 125(41), 12466-12467.

8. Li, Y., Li, H., Li, S., & Huang, X. (2007). Synthesis of high-quality CdSe quantum dots in aqueous solution. Chemical Communications, (44), 4627-4629.

9. Carleton College. (2015). Transmission electron microscopy (TEM) of nanoparticles. Retrieved from https://serc.carleton.edu/details/images/180093.html

10. Chen, X., & Yoon, J. (2021). Recent advances in fluorescent and colorimetric probes for latent fingerprint detection. Wiley Interdisciplinary Reviews: Forensic Science, 3(4), e1475.

11. Wang, M., Li, M., Yu, A., Wu, J., & Mao, C. (2015). Rare Earth Fluorescent Nanomaterials for Enhanced Development of Latent Fingerprints. ACS applied materials & interfaces, 7 51, 28110-5 .

Questions?
